# ﻿Review of *Ophioplinthaca* Verrill, 1899 (Echinodermata, Ophiuroidea, Ophiacanthidae), description of new species in *Ophioplinthaca* and *Ophiophthalmus*, and new records from the Northwest Pacific and the South China Sea

**DOI:** 10.3897/zookeys.1099.76479

**Published:** 2022-05-11

**Authors:** Hasitha Nethupul, Sabine Stöhr, Haibin Zhang

**Affiliations:** 1 Institute of Deep-sea Science and Engineering, Chinese Academy of Sciences, CAS, 57200 Sanya, China Institute of Deep-sea Science and Engineering, Chinese Academy of Sciences Sanya China; 2 University of Chinese Academy of Sciences, Beijing 100039, China University of Chinese Academy of Sciences Beijing China; 3 Swedish Museum of Natural History, Dept of Zoology, Box 50007, 10405 Stockholm, Sweden Swedish Museum of Natural History, Dept of Zoology Stockholm Sweden

**Keywords:** COI, cold seep, molecular phylogeny, morphology, seamounts, SEM, taxonomy

## Abstract

The ophiuroid genus *Ophioplinthaca* is well characterized by the deep incisions in the disc. Prior to this study, it contained 32 accepted species, but species limits and geographic distributions were not well understood. The manned submersible vehicle ‘Shenhaiyongshi’ was used to collect ophiuroid specimens from the deep-sea seamounts and cold seeps in the South China Sea and Northwest Pacific at 602–3600 m depth, during 2018 to 2020. The genus *Ophioplinthaca* was reviewed using both morphological data and a phylogenetic analysis, based on COI sequences. The taxonomic status of the genus *Ophiophthalmus* Matsumoto, 1917, a junior homonym of *Ophiophthalmus* Fitzinger, 1843 (a reptile) was clarified by proving prevailing usage of the ophiuroid name. A total of eight species were identified, including two new species, described as*Ophioplinthacabrachispina***sp. nov.** and *Ophiophthalmusserratus***sp. nov.**, and two new records. The new species are characterized by unique features of the arm skeletons. Tabular keys to all *Ophioplinthaca* and *Ophiophthalmus* species are provided. Interspecific and intraspecific genetic distance of *Ophioplinthaca* species ranged from 2.32% to 19.72%, and from 0.26% to 0.90%, respectively. The data suggest that species of the genus *Ophioplinthaca* are more widely spread around the Northwest Pacific region deep-sea seamounts than previously known.

## ﻿Introduction

The ophiuroid family Ophiacanthidae Ljungman, 1867 is one of the largest and diverse families in the order Ophiacanthida, containing 239 accepted species within 15 genera to date (Paterson 1985; Martynov 2010a; Martynov et al. 2015; Stöhr et al. 2021). In the present study, we focused on the genera *Ophioplinthaca* Verrill, 1899 and *Ophiophthalmus* Matsumoto, 1917. *Ophioplinthaca* can easily be distinguished from other genera by deep incisions in the disc that create distally enlarged wedge-shaped lobes (Verrill 1899; O’Hara and Stöhr 2006). A total of 32 accepted species are included in the genus *Ophioplinthaca*, and most of them have been recorded from the Indo-Pacific Ocean (OBIS 2021; Stöhr et al. 2021). Recent studies suggested *Ophioplinthaca* species were dominant megafauna on seamounts from the Northwest Pacific region (Cho and Shank 2010; Chen et al. 2021a; Na et al. 2021). However, species diversity and geography of *Ophioplinthaca* species are still not fully understood due to limited collecting efforts in this area (Cho and Shank 2010; Yesson et al. 2011; Chen et al. 2021a, b; Na et al. 2021). Previous morphological studies reported that *Ophioplinthaca* species were difficult to separate due to complex intraspecific morphological variation (O’Hara and Stöhr 2006; Chen et al. 2021a, b; Na et al. 2021).

The genus *Ophiophthalmus* was created by Matsumoto (1917) to accommodate particular species that at the time were placed in the genera *Ophiomitra*, *Ophiomitrella*, and *Ophiacantha*, but currently only four species are included in this genus. Paterson (1985) considered *Ophiophthalmus*as an invalid junior homonym of a reptilian genus described by Fitzinger (1843), without proposing a replacement name. Therefore, the taxonomic status of *Ophiophthalmus* will be clarified herein.

This study covers deep waters around the Northwest Pacific region near southwest Guam Island, and in the South China Sea (Xisha Islands and Haima cold seep). Here, we present an account of the ophiuroid species collected. Our goal is to present a diagnosis of the morphological features of these species, combined with molecular details, to complement the limited original descriptions and the lack of figures in the literature. We present comprehensive tabular keys for all species within the genera *Ophioplinthaca* and *Ophiophthalmus*. Two new species, one in *Ophioplinthaca* and one in *Ophiophthalmus*, are described, and six species of *Ophioplinthaca* are redescribed, including two new records from the Northwest Pacific region, all richly illustrated. These species live on seamounts and cold seeps, and this study adds to the known diversity in these unique habitats to better understand ophiuroid distribution and biogeography.

## ﻿Materials and methods

### ﻿Sample collecting

**Figure 1.**
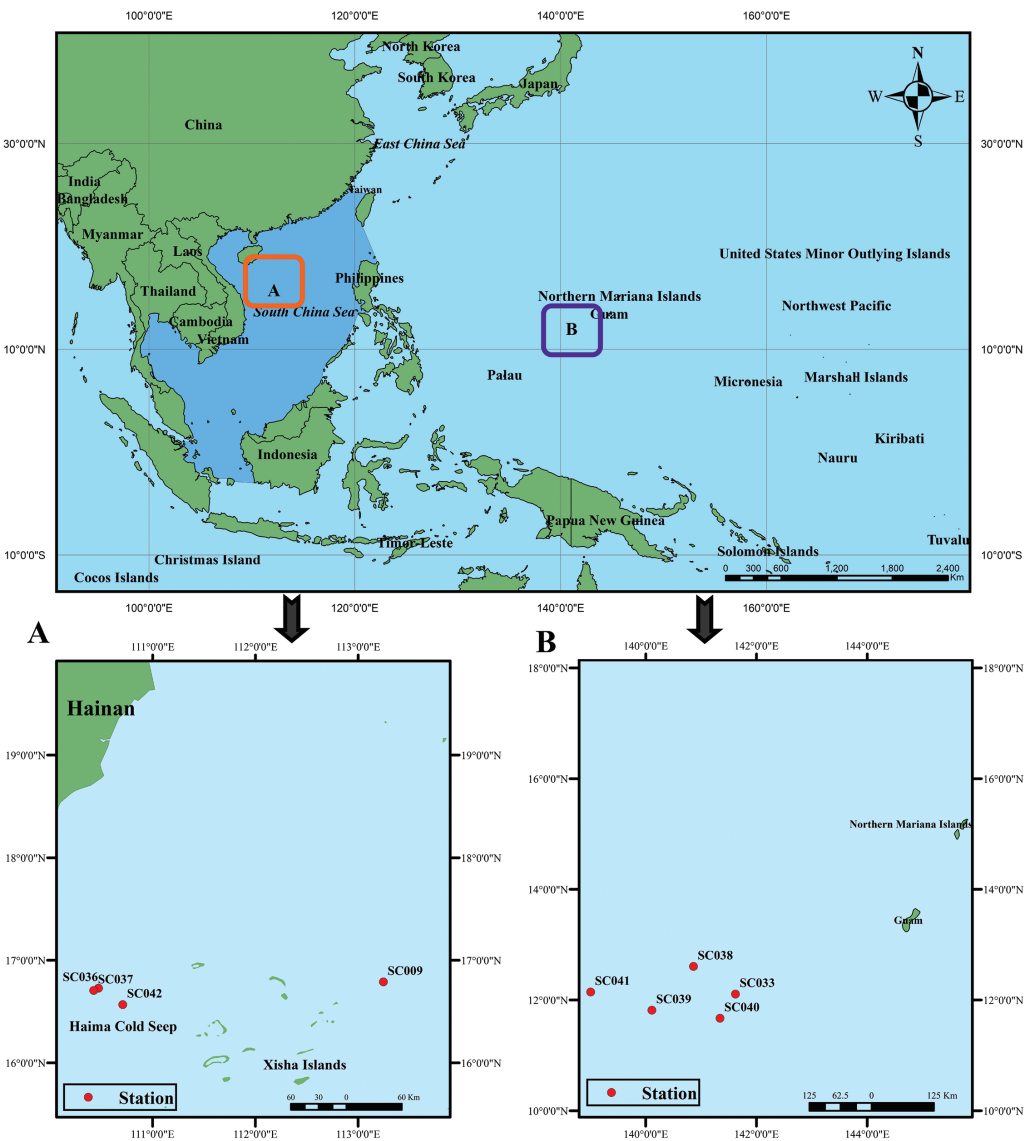
Collecting stations in this study **A** South China Sea (Xisha Islands and Haima cold seep) **B** Northwest Pacific region (Southwest of Guam island). Source: International Hydrographic Organization and Sieger 2012.

The manned submersible vehicle ‘Shenhaiyongshi’ was used to collect samples for this study on a seamount near Xisha Islands and on the Haima cold seep in the South China Sea, as well as on a seamount southwest of Guam Island (Fig. 1). Most of the specimens were frozen without preservation fluid, then transported to the Institute of Deep-sea Science and Engineering, Chinese Academy of Sciences (CAS), Sanya, China, for further analysis. The samples were sorted and the species identified using available literature (Thomson 1877; Lyman 1878, 1882, 1883; Koehler 1904, 1922, 1930, 1897; Clark 1900, 1911, 1915, 1939; Matsumoto 1917; Clark 1949; Mortensen 1933; John and Clark 1954; Cherbonnier and Sibuet 1972; Guille 1981; O’Hara and Stöhr 2006; Chen et al. 2021a, b; Na et al. 2021) and by molecular analysis.

### ﻿Morphological analysis

Specimens were photographed through a dissecting stereo microscope (OLYMPUS SZX7) to identify external morphological characters. Arm skeletons were photographed by a scanning electron microscope **(SEM)** Phenom ProX. Arm skeletal elements were prepared by dissolving the soft tissue in undiluted NaOCl. The excess NaOCl in skeletal elements (ossicles) was removed by repeated flushing with distilled water. After drying, the ossicles were mounted on a stub, using ethanol dissolvable carbon tapes. Holotypes, paratypes and all other newly recorded specimens were deposited at the Institute of Deep-sea Science and Engineering **(CAS)**, Sanya, China. The terms used to describe ophiuroids follow previous authors (Martynov 2010a, b; Stöhr 2011, 2012; O’Hara et al. 2017; Hendler 2018; Stöhr and O’Hara 2021).

Type material and one other specimen of *Ophioplinthacalithosora* (H. L. Clark, 1911) were examined from digital photographs.

### ﻿Molecular analysis

DNA of identified specimens was extracted by using the TIANamp Marine Animals DNA kit (TianGen, Beijing) following the manufacturer’s protocol. We sequenced cytochrome c oxidase I (COI) partial genes for phylogenetic analysis by amplifying COIceF (5´- ACTGCCCACGCCCTAGTAATGATATTTTTTATGGTNATGCC-3´) and COIceR(5´-TCGTGTGTCTACGTCCATTCCTACTGTRAACATRTG-3´) COI primer set, with an initial denaturation at 95 °C for 3 min, followed by 40 cycles of denaturation at 94 °C for 45 s, annealing temperature at 51 °C to 55 °C for 70 s, and extension at 72 °C for 80 s; and a final extension at 72 °C for 5 min as a suitable PCR cycle (Hoareau and Boissin 2010). Total PCR mixture was 50 μL volume, containing 25 μL Premix Taq with 1.25 U Taq, 0.4 mM of each dNTP and 4 mMMg2+ (Ex Taq version, Takara, Dalian, China), 0.5 μM each of the primers and approximately 100 ng template DNA. PCR product quality was determined by electrophoresis using a 1.0% agarose gel and the NanoDrop 1000 (Thermo Scientific, Waltham, MA, USA). PCR products were sequenced in both directions on ABI3730 DNA Analyzer, and all new sequences were deposited at NCBI GenBank.

We constructed a maximum likelihood (ML) phylogenetic tree to represent the family Ophiacanthidae by adding ten species from our collection and an additional 11 sequences from NCBI GenBank (Table 1). As outgroup we used *Ophiomyxabrevirima* H. L. Clark, 1915 and *Ophiomyxaanisacantha* H. L. Clark, 1911. All sequences were aligned using the Clustal W algorithm in MEGA X. The best-fit substitution model of the COI gene in the ML trees was T92 + G + I model (Tamura 3-parameter model + Gamma distributed with invariant sites), and estimated by the “Find Best DNA/Protein Models” Option of MEGA X. A phylogenic tree was reconstructed for the partial COI gene by using the maximum likelihood bootstrap method. The ML analysis was run with MEGA X, and ML trees were constructed, including 1,000 bootstrap replicates (Kimura 1980; Thompson et al. 1994; Kumar et al. 2016, 2018). The genetic distances with standard error of specimen groups were analyzed according to the Kimura 2-parameter model with performing 1.000 bootstrap replications (Kimura 1980).

**Table 1.** Localities, voucher information, and GenBank accession numbers for all specimens used in this study.

Species	Locality	Voucher number	COI
***Ophioplinthaca* sp.**	Mariana Trench: Southwest of Guam island	IDSSE-EEB-SW0108	OK043831
***Ophioplinthacadefensor***	Mariana Trench: Southwest of Guam island	IDSSE-EEB-SW0112	OK043836
***Ophioplinthacadefensor***	Northwest Pacific Ocean: Caiwei Guyot	RSIO410611	MT025778
***Ophioplinthacaathena***	Mariana Trench: Southwest of Guam island	IDSSE-EEB-SW0110	OK043833
***Ophioplinthaca* sp.**	Northwest Pacific Ocean: St. RC-ROV08	RSIO56058	MW284981
**Ophioplinthacacf.lithosora**	South China Sea: Xisha islands	IDSSE-EEB-SW0111	OK043834
***Ophioplinthacaglobata***	Papua New Guinea	MNHN BP32	KU895134
***Ophioplinthacasemele***	Northwest Pacific Ocean: St. RC-ROV08	RSIO56057	MW284980
***Ophioplinthacasemele***	Mariana Trench: Southwest of Guam island	IDSSE-EEB-SW0113	OK043835
***Ophioplinthacaplicata***	Australia: Tasman Sea	MV F144758	EU869989
***Ophioplinthacaplicata***	New Zealand	MV F188868	KU895133
***Ophioplinthacagrandisquama***	Northwest Pacific Ocean: St. RC-ROV05	RSIO56060	MW284982
***Ophioplinthacaamezianeae***	Mariana Trench: Southwest of Guam island	IDSSE-EEB-SW0109	OK043832
***Ophioplinthacabrachispina* sp. nov.**	Mariana Trench: Southwest of Guam island	IDSSE-EEB-SW0106	OK043829
***Ophioplinthacabrachispina* sp. nov.**	Mariana Trench: Southwest of Guam island	IDSSE-EEB-SW0107	OK043830
***Ophiophthalmuscataleimmoidus***	Canada: British Columbia, Kyoquot Sound	RBCM EC00208	HM542946
***Ophiophthalmusnormani***	Canada: British Columbia, Kyoquot Sound	RBCM EC00186	HM542947
***Ophiophthalmusserratus* sp. nov.**	South China Sea: Haima cold seep	IDSSE-EEB-SW0136	OK043837
***Ophiophthalmusserratus* sp. nov.**	South China Sea: Haima cold seep	IDSSE-EEB-SW0137	OK043838
***Ophiomyxabrevirima***	New Zealand	MVF95868	KU895170
***Ophiomyxaanisacantha***	Japan: Sagami Sea	NSMT E-6269	AB758822

The following abbreviations are used in the text, tables, and figures:

**ars** arm spine;

**as** adoral shield;

**ASE** arm segment;

**ass** adoral shield spine;

**COI** Cytochrome C oxidase subunit 1;

**D** dorsal;

**DAP/dap** dorsal arm plate;

**DAS/das** dorsal arm spines;

**de** depression;

**dist** distal;

**dl** dorsal lobe;

**ds** disc spine;

**dsc** disc scale;

**gra** granules;

**gs** genital slit;

**IDSSE** Institute of Deep-sea Science and Engineering;

**j** jaw;

**lac** lateral ambulacral canal;

**lap** lateral arm plate;

**LOP** lateral oral papillae;

**m** madreporite;

**ML** Maximum Likelihood;

**mo** muscle opening;

**msv** manned submersible vehicle;

**no** nerve opening;

**NSMT**National Science Museum, Tokyo;

**os** oral shield;

**pb** podial basin;

**prox** proximal;

**ri** ridge;

**RS/rs** radial shield;

**th** thorns;

**TS/ts** tentacle scale;

**v** ventral;

**VAP/vap** ventral arm plate;

**VAS/vas** ventral arm spines;

**vl** ventral lobe;

**VMT** ventralmost tooth;

**vs** volute-shape;

**USNM**United States National Museum, Smithsonian Institution.

## ﻿Results

Seven species of *Ophioplinthaca* were identified, among them one new to science, and all are described below. One specimen was identified as belonging to *Ophiophthalmus* and is described as a new species. One unidentified specimen of *Ophioplinthaca* is described, but not assigned to a name pending further investigations of variability within the genus. A tabular key to all species of *Ophioplinthaca* is provided in Table 3, to the species in *Ophiophthalmus* in Table 4.

### ﻿Molecular phylogenetic analysis

**Figure 2.**
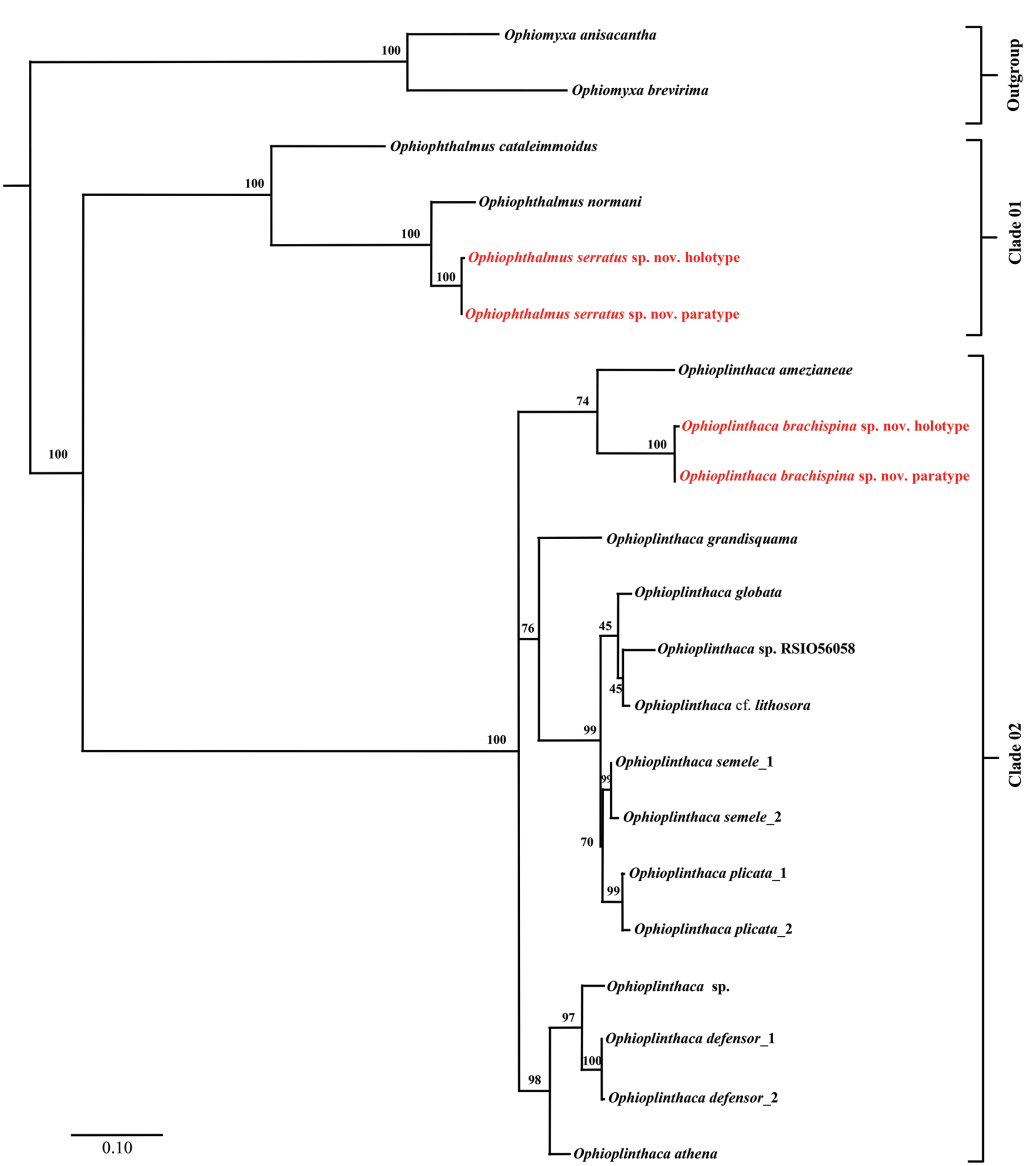
Maximum likelihood (ML) tree of *Ophioplinthaca* and *Ophiophthalmus*, based on partial COI sequences (bootstrap support values were generated with rapid bootstrapping algorithm for 1,000 replicates; red = new species).

A total of 21 COI sequences trimmed to 581 bp were obtained after removing ambiguous aligned sites and successfully reconstructing a genera *Ophioplinthaca* and *Ophiophthalmus*ML tree (Fig. 2).

Two main clades were detected within the ML Tree (clade 01: genus *Ophiophthalmus*); clade 02: genus *Ophioplinthaca*). Average mean genetic distance of Ophiacanthidae was 21.74 ± 2.79% SE (19 specimens), and maximum value between two genera was 46.09 ± 4.81% SE. Overall average mean genetic distance of COI within *Ophioplinthaca* was 11.85 ± 1.70% SE (15 specimens). Interspecies and intraspecies genetic distance range among *Ophioplinthaca* species were 2.32–19.72% and 0.26–0.9% respectively. Overall average mean genetic distance of COI among *Ophiophthalmus* was 12.99 ± 1.76% SE (4 specimens), and interspecies genetic distance ranged between 7.06–21.20% (Table 2).

**Table 2.** *Ophioplinthaca* and *Ophiophthalmus*, pairwise distance values based on 581 bp mitochondrial COI sequences, calculated using the Kimura 2-parameter method with 1,000 bootstrap replicates (values in blue color represent Standard Error).

No.	Species	P-distance (%)																				
		1	2	3	4	5	6	7	8	9	10	11	12	13	14	15	16	17	18	19	20	21
**1**	*Ophioplinthaca* sp.		0.90%	1.15%	1.16%	2.13%	1.64%	1.86%	1.92%	1.59%	1.91%	1.60%	2.01%	2.01%	1.97%	2.02%	3.60%	3.80%	3.12%	3.11%	3.47%	3.54%
**2**	*Ophioplinthacadefensor*_1	4.27%		0.26%	1.17%	2.11%	1.63%	1.81%	1.97%	1.60%	1.89%	1.60%	1.68%	1.94%	1.85%	1.89%	3.70%	3.97%	3.24%	3.23%	3.37%	3.52%
**3**	*Ophioplinthacadefensor*_2	4.34%	0.26%		1.45%	2.26%	2.04%	2.39%	1.95%	1.95%	1.97%	2.01%	1.81%	2.41%	2.36%	2.43%	4.25%	4.70%	4.68%	4.64%	4.76%	4.75%
**4**	*Ophioplinthacaathena*	6.73%	7.09%	7.20%		1.94%	1.54%	1.76%	1.71%	1.50%	1.70%	1.55%	1.84%	1.78%	1.81%	1.86%	3.69%	3.85%	3.28%	3.27%	3.40%	3.51%
**5**	*Ophioplinthaca* sp. RSIO56058	15.13%	15.34%	15.59%	12.50%		1.01%	1.41%	1.29%	1.22%	1.39%	1.36%	1.98%	2.23%	2.16%	2.21%	3.90%	4.33%	4.70%	4.68%	4.54%	4.32%
**6**	Ophioplinthacacf.lithosora	13.34%	13.29%	13.89%	11.47%	4.17%		0.73%	1.03%	0.85%	1.04%	0.95%	1.84%	1.92%	1.84%	1.88%	3.86%	4.33%	3.64%	3.66%	3.60%	3.66%
**7**	*Ophioplinthacaglobata*	13.06%	12.20%	11.77%	11.16%	5.21%	2.32%		1.31%	0.96%	1.33%	1.20%	2.04%	2.15%	2.02%	2.07%	4.28%	4.69%	3.63%	3.62%	3.95%	4.04%
**8**	*Ophioplinthacasemele*_1	12.97%	13.81%	13.24%	10.28%	5.81%	4.15%	4.43%		0.43%	1.01%	1.11%	1.80%	2.48%	2.34%	2.40%	4.25%	4.56%	4.80%	4.81%	4.74%	4.52%
**9**	*Ophioplinthacasemele*_2	13.15%	13.32%	13.26%	11.26%	5.48%	3.92%	4.00%	0.76%		0.88%	0.80%	1.74%	1.98%	1.82%	1.86%	3.97%	4.29%	3.47%	3.47%	3.47%	3.58%
**10**	*Ophioplinthacaplicata*_1	13.88%	13.80%	13.57%	11.40%	6.76%	4.39%	5.08%	3.87%	3.20%		0.45%	1.72%	2.44%	2.25%	2.28%	4.12%	4.38%	4.74%	4.77%	4.15%	4.06%
**11**	*Ophioplinthacaplicata* _2	13.34%	13.51%	14.18%	12.05%	6.74%	5.01%	5.95%	4.67%	3.54%	0.90%		1.79%	2.04%	1.91%	1.94%	4.12%	4.48%	3.68%	3.69%	3.31%	3.48%
**12**	*Ophioplinthacagrandisquama*	13.96%	10.46%	10.79%	11.62%	13.89%	12.14%	10.26%	11.43%	11.27%	11.25%	12.08%		2.50%	2.31%	2.36%	4.22%	4.33%	4.47%	4.44%	4.35%	4.48%
**13**	*Ophioplinthacaamezianeae*	16.84%	15.90%	15.96%	14.17%	16.99%	16.45%	16.04%	18.62%	17.81%	19.72%	18.21%	19.37%		1.70%	1.68%	3.59%	3.78%	3.16%	3.14%	3.59%	3.58%
**14**	*Ophioplinthacabrachispina* sp. nov. holotype	17.35%	15.96%	17.44%	14.76%	15.80%	15.35%	15.43%	16.97%	15.37%	17.66%	16.40%	18.83%	12.66%		0.25%	3.96%	4.15%	3.38%	3.35%	3.80%	3.98%
**15**	*Ophioplinthacabrachispina* sp. nov. paratype	17.68%	16.25%	17.80%	15.03%	16.13%	15.42%	15.53%	17.32%	15.45%	17.68%	16.27%	19.17%	12.46%	0.35%		3.91%	4.10%	3.36%	3.33%	3.86%	4.03%
**16**	*Ophiophthalmuscataleimmoidus*	35.83%	36.53%	39.34%	35.96%	38.04%	39.02%	34.92%	41.28%	39.91%	41.56%	41.75%	41.45%	35.60%	39.28%	39.24%		2.52%	2.54%	2.56%	3.96%	3.56%
**17**	*Ophiophthalmusnormani*	37.88%	40.61%	44.94%	37.99%	41.88%	43.15%	37.96%	43.18%	42.77%	43.61%	45.13%	43.34%	37.52%	40.43%	40.38%	21.20%		1.37%	1.34%	3.62%	3.51%
**18**	*Ophiophthalmusserratus* sp. nov. holotype	35.29%	36.45%	44.60%	35.71%	45.98%	41.04%	34.51%	45.59%	39.18%	47.62%	41.61%	45.15%	34.14%	36.18%	36.04%	20.86%	7.31%		0.24%	2.99%	3.13%
**19**	*Ophiophthalmusserratus* sp. nov. paratype	34.99%	36.15%	44.08%	35.41%	45.50%	41.04%	34.51%	46.09%	39.18%	48.09%	41.61%	44.68%	33.56%	35.58%	35.43%	21.17%	7.06%	0.35%		3.00%	3.15%
**20**	*Ophiomyxabrevirima*	41.60%	40.93%	47.22%	41.42%	45.76%	41.96%	39.47%	47.33%	40.93%	43.09%	38.75%	44.10%	41.77%	45.04%	45.07%	39.60%	38.90%	35.26%	35.54%		2.05%
**21**	*Ophiomyxaanisacantha*	41.81%	41.07%	46.86%	40.25%	43.58%	40.99%	38.67%	44.39%	40.64%	41.50%	39.95%	44.05%	41.43%	45.62%	46.31%	35.76%	35.91%	35.47%	35.77%	17.85%	

The new species, described morphologically below, were confirmed by the molecular analysis as separate from all other sequenced species (Fig. 2) and species identified by morphological characters were confirmed by the COI analysis.

### ﻿Taxonomic account

**Superorder Ophintegrida O’Hara, Hugall, Thuy, Stöhr and Martynov, 2017**

**Order Ophiacanthida O’Hara, Hugall, Thuy, Stöhr and Martynov, 2017**

**Suborder Ophiacanthina O’Hara, Hugall, Thuy, Stöhr and Martynov, 2017**

**Family Ophiacanthidae Ljungman, 1867**

#### Genus *Ophioplinthaca* Verrill, 1899

##### ﻿ Ophioplinthaca brachispina sp. nov.

Taxon classificationAnimaliaAmphilepididaOphiacanthidae

CD697DAC-9098-5A5B-B752-B5DF4EAB5A5E

http://zoobank.org/B225308A-59B8-431C-B9AF-1E4F729878D2

Figs 3 , 4 , 5

###### Material examined.

***Holotype*.** Northwest Pacific • 1 specimen; near Mariana Trench, Southwest of Guam Island, seamount; 11°49.09'N, 140°6.93'E; depth 2713 m; 23 October 2019; Collecting event: stn. SC039; Shenhaiyongshi msv leg; preserved in -80 °C; GenBank: OK043829; IDSSE-EEB-SW0106. ***Paratype*.** Northwest Pacific • 1 specimen; same data as for holotype; GenBank: OK043830; IDSSE-EEB-SW0107.

###### Diagnosis.

**Figure 3.**
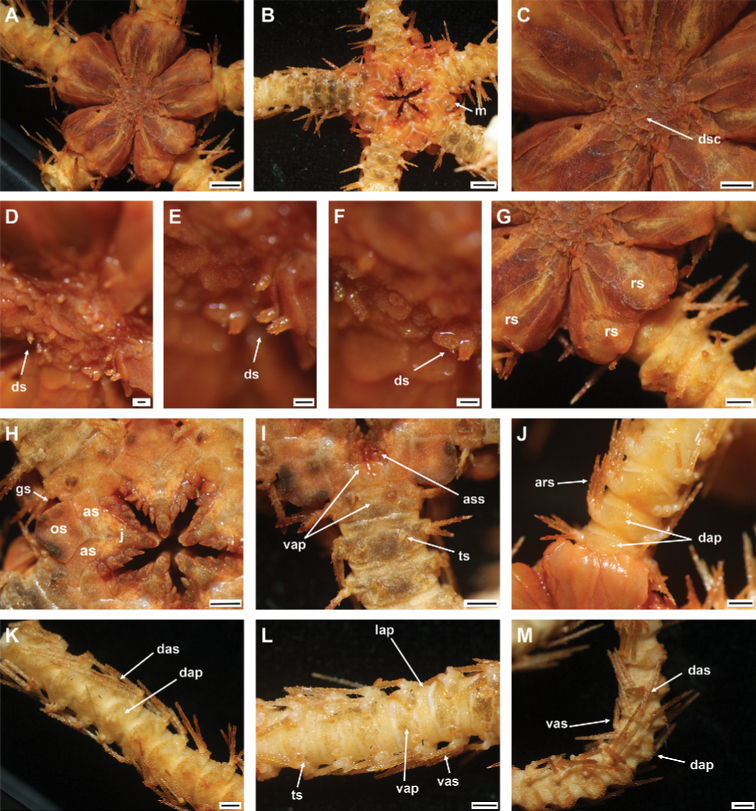
*Ophioplinthacabrachispina* sp. nov., holotype (IDSSE-EEB-SW0106) **A** dorsal disc **B** ventral disc **C** center of the disc **D–F** disc spines **G** radial shield **H** оral frame **I** ventral side of the arm base **J** dorsal side of the arm base **K** dorsal arm **L** ventral arm **M** lateral arm. Abbreviations: **ars** arm spine, **as** adoral shield, **ass** adoral shield spine, **dap** dorsal arm plate, **das** dorsal arm spine, **dp** disc plate, **gs** genital slit, **j** jaw, **lap** lateral arm plate, **m** madreporite, **os** oral shield, **rs** radial shield, **ts** tentacle scale, **vap** ventral arm plate, **vas** ventral arm spine. Scale bars: 2 mm (**A, B**); 1 mm (**C, G–M**); 200 µm (**D–F**).

Disc sub-circular and deeply incised interradially to nearly 1/4 disc radius (Fig. 3A). Disc scales irregular, variable in size, bearing disc spines in center of disc (Fig. 3C). Radial shields completely separated by large single disc scale (Fig. 3G). Oral shield as wide as long, pentagonal with pointed proximal end, curved lateral margins along adoral shields, truncated distal edge with straight to slightly angular lateral margins (Fig. 3H). Surface of arm plates along entire arm rough with small spines (Fig. 3K–M).

###### Holotype description.

Disc diameter 12 mm, arm base width 3 mm (Fig. 3).

***Disc*.** Disc sub-circular and deeply incised interradially to more than 1/3 disc diameter, creating five wedge-shaped lobes over each arm base in contrast to sunken center and interradii of disc (Fig. 3A, B). Disc scales irregular, variable in size, compact, and overlapping in center of disc (Fig. 3C). Most central disc scales bear disc spines/stumps (Fig. D–F). Disc scales increasingly enlarged from disc center to periphery, interradially and between radial shields (Fig. 3D–F). Disc spines in disc center 0.2 to 0.3 mm high, cylindrical to conical, pointed thorny or bifurcated tip. Disc spines at distal end of wedge-shaped lobes 0.1–0.2 mm high, conical, thorny, with pointed tip (Fig. 3D–F). Radial shields large, naked, roughly triangular, ~ 1/3 disc diameter in length, twice as long as wide, triangular proximal end, and smooth, truncated or slightly convex distal end. Radial shields on three of five lobes proximally separated, but distal ends connected. Radial shields on other two lobes completely separated by large single disc scale (Fig. 3A, G). Ventral disc covered by smaller scales than those on radial shields, and overlapped without bearing spines (Fig. 3B). Genital slits conspicuous and extending from oral shield to periphery of disc (Fig. 3H). Oral shield as wide as long, pentagonal with pointed proximal end, curved lateral margins along adoral shields, truncated distal edge with straight to slightly angular lateral margins (Fig. 3H). Madreporite similar to other oral shields, but with hydropore at lateral edge (Fig. 3B). Adoral shield 2.5 × as long as wide, with straight or slightly curved lateral margin, but near first ventral arm plate straight, and pair of shields proximally connected (Fig. 3H). Adoral shields enclose proximal edges of oral shield, and slightly separate oral shield from arm by connecting to lateral arm plate of first arm segment (Fig. 3H, I). Jaw triangular, large, and longer than wide, bearing one slightly blunt, wide, and large ventralmost tooth and three or four spiniform lateral oral papillae (Fig. 3H). Proximalmost one or two lateral oral papillae spine-like pointed, rugose, and distalmost lateral oral papillae with shorter and rounded base with more or less pointed tip (Fig. 3H). One adoral shield spine, situated at lateral margin of adoral shield in mouth angle, slightly similar to distalmost lateral oral papilla, but with blunt tip (Fig. 3I). Cluster of small granules visible between distal end of jaw and proximal end of first ventral arm plate (Fig. 3I). Usually, cluster of granules covered by adoral shield spine (Fig. 3H).

***Arms*.** Five moniliform arms with rough plates. Dorsal arm plates longer than wide, slightly separated, straight to slightly convex distal end, triangular proximal end, with curved lateral margins on first few proximal arm segments, but as long as wide, fan-shaped, and widely separated on middle to distal half of arm (Fig. 3J, K). Dorsal arm plate with dense rough surface and short spines (Fig. 3K, M). First ventral arm plate rectangular to slightly trapezoid, as wide as long, straight proximal end, and distal end without rough surface (Fig. 3I). Second ventral arm plate trapezoid to slightly pentagonal, as wide as long, triangular proximal end, straight distal end, concave and diverging lateral edges, and contiguous with first ventral arm plate (Fig. 3I). The following ventral arm plates two or three times as wide as long, with obtuse proximal end, slightly wavy proximolateral margins, curved lateral angles, straight distal end, and widely separated (Fig. 3L). All ventral arm plates except first one with dense rough surface (Fig. 3I, L). Lateral arm plates meeting above and below, with dense rough surface and short spines (Fig. 3K–M). Up to five arm spines (Fig. 3M). Three or two dorsal arm spines, three arm segments in length, thorny, lateral margins with row of tall sharp thorns, apex truncated or bluntly rounded (Fig. 3K–M). Two ventral arm spines, one to two arm segments in length, pointed, thorny, rugose. Proximal arm segments bear five arm spines, distalwards decreasing to four beyond middle section of the arm (Fig. 3K–M). First tentacle pore covered by two oval, rough tentacle scales (Fig. 3I). The following tentacle pore covered by scale half as long as ventral arm plate, blunt to pointed tip with thorny surface (Fig. 3L). Tentacle scales on middle to distal half of arm decreasing in size, small, more pointed, leaf-like, with thorns.

***Color*.** In live specimen, orange-brown disc, and arm spines, but arms pale brown (Fig. 3).

###### Ossicle morphology of paratype.

**Figure 4.**
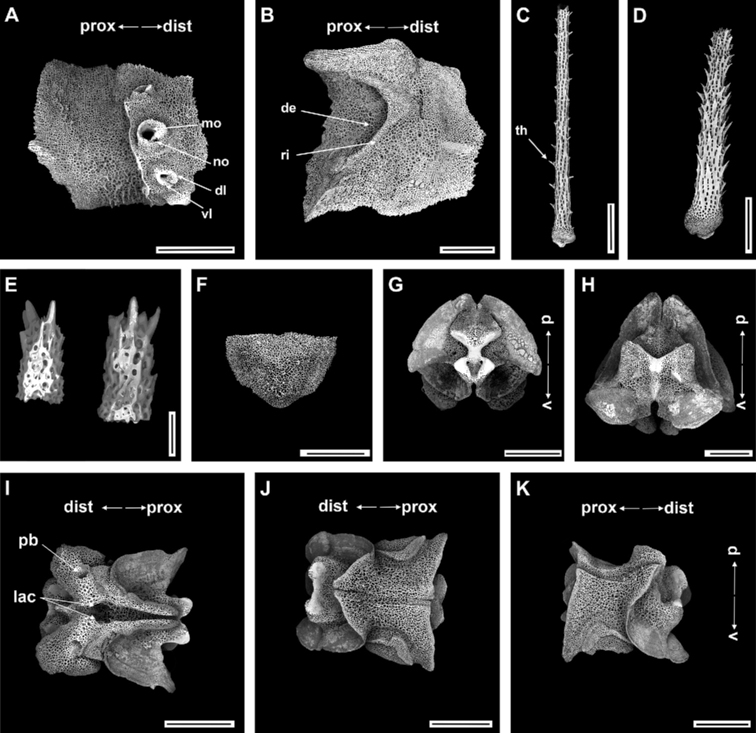
*Ophioplinthacabrachispina* sp. nov., paratype (IDSSE-EEB-SW0107) **A, B** lateral arm plate **C** dorsal arm spine **D** ventral arm spine **E** disc spine **F** dorsal arm plate **G–K** vertebrae **G** proximal view **H** distal view **I** ventral view, **J** dorsal view, **K** lateral view. Abbreviations: **d** dorsal, **de** depression, **dist** distal, **dl** dorsal lobe, **lac** lateral ambulacral canals, **mo** muscle opening, **no** nerve opening, **pb** podial basin, **prox** proximal, **ri** ridge, **th** thorns, **v** ventral, **vl** ventral lobe. Scale bars: 800 µm (**A, C, F–G, I–K**); 500 µm (**B, E, H**); 100 µm (**D**).

Arm spine articulations well developed and placed at slight angle to distal edge of lateral arm plate (Fig. 4A). Volute-shaped perforated lobe forms dorsal and distal part of articulation, but reduced in dorsalmost one (Fig. 4A). Arm spine articulating structures with large muscle opening and small nerve opening in second articulation, decreasing significantly in size ventralwards (Fig. 3A). Ventral half of lateral arm plate surface covered by conspicuous thorns, inner side with depression, a continuous ridge, and a prominent knob close to ventral edge forming vertebral articulations, shaped like a broad, nose-shaped beak (Fig. 4A, B). Dorsal arm spine laterally compressed, thorny, and several longitudinal rows of perforations with widely spaced tall thorns (Fig. 4C). Entire ventral arm spine surface covered with slightly longer thorns, with blunt apex (Fig. 4D). Disc spines 0.2–0.3 mm high, cylindrical, pointed thorny or bifurcated tip (Fig. 4E). Dorsal arm plate triangular, as long as wide, with rugose surface (Fig. 4F). Vertebrae with streptospondylous articulating structures, short, broad podial basin at proximal end and narrow small distal end (Fig. 4G–K). Dorsal end of vertebrae distally triangular and proximally flattened with longitudinal groove along midline (Fig. 4J). Ventral side of vertebrae with broad ambulacral groove (Fig. 4I–K).

###### Paratype variations.

**Figure 5.**
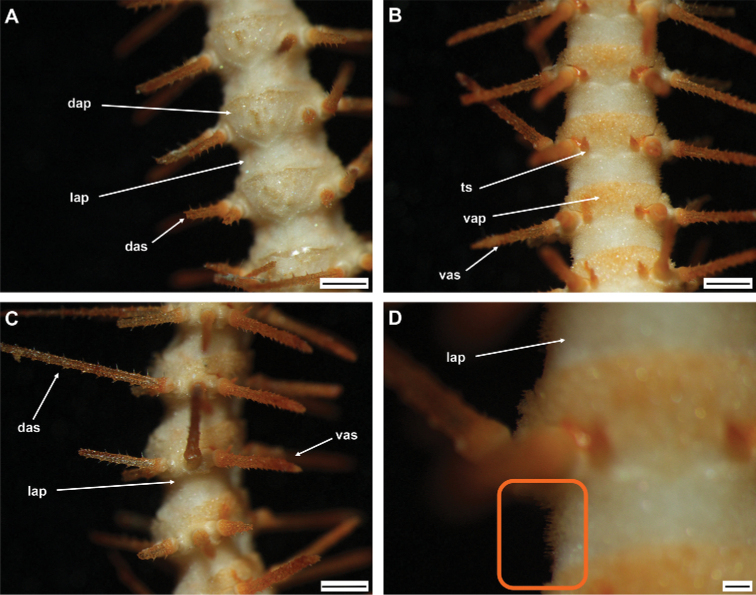
*Ophioplinthacabrachispina* sp. nov., paratype (IDSSE-EEB-SW0107) **A** dorsal arm **B** ventral arm **C** lateral arm **D** lateral arm plate (small thorns on the lateral arm plate surface shown in the orange rectangle). Abbreviations: **dap** dorsal arm plate, **das** dorsal arm spine, **lap** lateral arm plate, **ts** tentacle scale, **vap** ventral arm plate, **vas** ventral arm spine. Scale bars: 1 mm (**A–C**); 200 µm (**D**).

One specimen from same location as holotype, but badly damaged due to rough handling. Therefore, only small disc part with arms present. Possibly smaller than holotype according to size of arms (arm base width 1.5–2 mm). Arm characters similar to holotype, but spines slightly thinner, and denser compared to holotype (Fig. 5A–D).

###### Distribution.

2713 m depth, Northwest Pacific, near Mariana trench, Southwest of Guam Island.

###### Etymology.

Species name derived from a combination of two Latin words, *brachium* (arm), *spina* (spine) referring to the unique rough arm surface with spines.

###### Remarks.

Deep interradial incisions into the disc, which are lined distally by enlarged disc scales are the main delimiting character of the genus *Ophioplinthaca* from other genera within the family Ophiacanthidae. *Ophioplinthacabrachispina* sp. nov. showed similar morphological characters to many other *Ophioplinthaca* species. However, *O.brachispina* sp. nov. can easily be distinguished from congeners by the rough thorny surface on the arm plates and additionally by the number of arm spines, disc spines, and tentacle scale (Table 3). *Ophioplinthacabrachispina* sp. nov. is the only *Ophioplinthaca* species with a rough surface with thorns on the whole arm.

**Table 3.** Tabular key to all species of the genus *Ophioplinthaca*. Abbreviations: **ASE** arm segment, **ASS** Adoral shield spine, **DAP** dorsal arm plate, **DAS** dorsal arm spines, **L** length, **LOP** lateral oral papillae, **VAP** ventral arm plate, **VAS** ventral arm spines, **VMT** ventralmost tooth, **W** width.

Species	No. of arm spines	Radial shields	Oral frame	Tentacle scale	Dorsal arm plate (DAP), and ventral arm plate (VAP)	Arm spine shape and length	Disc spines	References
*Ophioplinthacaabyssalis* Cherbonnier & Sibuet, 1972	up to 7	separated proximally but connected at distal ends	4–5 LOP, spiniform, 1 VMT	1^st^ pore 2, then 1, elongated, blunt	VAP-separated, DAP 1–3 have few spines and contiguous then separated	thorny surface	long, conical	Cherbonnier and Sibuet (1972)
*Ophioplinthacaamezianeae* O’Hara & Stöhr, 2006	up to 10	separated, L = 2W	up to 4–5 LOP, spiniform, 1 VMT	1^st^ pore 2–3, 2^nd^ pore 1–2, then 1, spiniform with rounded base, tapering sharply or tip covered in irregular thorns	separated	covered with conspicuous thorns	long, rounded base, with spinelets on lateral surface	O’Hara and Stöhr (2006), this study
*Ophioplinthacaathena* A.H. Clark, 1949	up to 5	separated proximally, connected at distal ends, L = 4W	4 LOP, pointed, flattened, larger, equal in size, 1 VMT	1^st^ pore 2, then 1, oval, pointed, beyond 3^rd^ pore slender spiniform	VAP on 1^st^ASE contiguous with next, then separated, DAP contiguous (terminal portion slightly separated)	1^st^DAS smooth, 2 × ASE length; 2^nd^DAS, 4 × ASE length; shorter thorny VAS	elongated with thorny tip, at periphery with smooth tip	A. H. Clark (1949), this study
*Ophioplinthacabrachispina* sp. nov.	up to 5	separated by large single disc scale or connect at distal ends	4 LOP, pointed, distal one with wider flat edge, 1 VMT, ASS covered by cluster of granules	1^st^ pore 2, half as long asVAP, terminally spiniferous, oval on base of arm, then slightly pointed along arm	DAP and VAP separated, thorny surface	DAS with distal lateral thorns; VAS ≈ 1–2 × ASE length, pointed, thorny, rugose	bifid or thorny pointed tip, cylindrical	this study
*Ophioplinthacabythiaspis* (H. L. Clark, 1911)	up to 6	separated, L = 4W	4–5 LOP, equal in height, distally slightly wider, 1 VMT	oval to bottle-shaped, as long asVAP	VAP separated beyond 3 ASE	DAS smooth, VAS with thorny surface	conical or more rounded with thorns at tip	H. L. Clark (1911, 1915), O’Hara and Stöhr (2006)
*Ophioplinthacacarduus* (Lyman, 1878)	up to 6	separated	4–5 LOP, first 3–4 conical, blunt tip, distalwards flat, spearhead-shaped, small, pointed, 1 VMT, curved adoral shield	strongly thorny, after 1^st^ pore, with 1 or 2 lateral thorns	VAP separated, DAP on 1–3 × ASE contiguous, then separated	stout, cylindrical, glassy, blunt, very thorny	short, stout stump with thorny tip	Lyman (1878)
*Ophioplinthacachelys* (C. W. Thomson, 1877)	up to 6	separated, deeply sunken	5 LOP, first 3–4 conical, blunt tip, 1 VMT	large and flat	VAP and DAP separated	cylindrical, glassy, blunt, very thorny	cylindrical, thorny or smooth, thorny tip, spines dense in disc center.	C. W. Thomson (1877)
*Ophioplinthacacitata* Koehler, 1904	up to 9	separated L = 3–4W	3–4 LOP, blunt, distal one has wider flat edge, 1 VMT	oval to elliptical	DAP contiguous, except first few ASE, VAP contiguous	DAS ≈ 4 × ASE length, VAS short, thorny, hollow	cylindrical, terminal crown of thorns	Koehler (1904), O’Hara and Stöhr (2006)
*Ophioplinthacaclothilde* A.H. Clark, 1949	up to 7 at arm base then 5	contiguous, ovoid, about half as long as broad	4 LOP, pointed, flattened, large, 1 VMT of equal size	1^st^ pore 2, then 1, leaf-like to narrow, sharply pointed with numerous spinelets at tip	VAP separated after 2 ASE, DAP separated	DAS ≈ 4 × ASE length, DAS and VAS with thorny surface, DAS longest	in disc center cylindrical base ending in 2–3 sub-crowns, at periphery short, stout with irregular crown of a dozen or more thorns	A. H. Clark (1949)
*Ophioplinthacacodonomorpha* (H. L. Clark, 1911)	up to 8	widely separated, small, convex proximally, as wide as long	3 LOP, 1 VMT	1^st^ pore 2, oval, conspicuously large, then oval with pointed tip	VAP at 1^st^ to 2^nd^ASE contiguous then separated, DAP barely contiguous	1–3 DAS smooth or thorny; VAS also smooth	minute rough granules	H. L. Clark (1911)
*Ophioplinthacacrassa* H. L. Clark, 1939	basally 4 then up to 5 or 7	distally connected, small, as wide as long, convex proximally	3 LOP, narrow, elongated, pointed, distalwards short and wide, 1 large VMT	1^st^ pore 2–3, blunt, 1mm long and wide, very thick, somewhat triangular, then less stout, slender pointed	VAP, DAP in first 1–2 ASE contiguous, then separated	short, stout, but fragile, thorny spines, DAS ≈ 2 × ASE length	low cylindrical tube	H. L. Clark (1939)
*Ophioplinthacadefensor* Koehler, 1930	up to 7 at arm base	separated proximally, connected distally	4 LOP, 1 VMT	1^st^ pore 1, long, and leaf-like then narrower with pointed tip	DAP and VAP contiguous	thorny surface, conspicuous thorns on DAS; DAS ≈ 3 × ASE, VAS ≈ 1 × ASE length	cylindrical with thorn at tip	Koehler (1930), Na et al. (2021), this study
*Ophioplinthacadipsacos* (Lyman, 1878)	up to 6	separated proximally, connected distally	4–5 LOP, pointed, flattened, ill-defined distalwards, 1 VMT	large, pointed with 1 or 2 microscopic thorns	VAP and DAP separated	long, slender, DAS 5–7 × ASE length, with conspicuous thorns, VAS 1½–2 × ASE length with thorny surface	short, stout, with thorny tip, at periphery smooth	Lyman (1878)
*Ophioplinthacaglobata* Koehler, 1922	up to 6	separated proximally, connected distally	3–4 LOP, bunt, distal one has wider flat edge, 1 VMT, ASS covered by small granules	1^st^ pore 2–3, as long asVAP, terminally spiniferous in larger specimen	DAP and VAP separated	thorny surface	cylindrical, bifid or tip mostly with 3 thorns	Koehler (1922, 1930), O’Hara and Stöhr (2006)
*Ophioplinthacagrandisquama* Chen, Na, & Zhang, 2021a	up to 7	contiguous, L = 1.5W	up to 3–4 LOP, spiniform, 1 or 2 VMT	1^st^ pore 1–2, only 1 in following ASE, long, thorny with thick base, tapering into blunt point	DAP contiguous, VAP separated	DAS thin with distal lateral thorns, up to 3 × ASE length; VAS short, blunt, and finely rugose	stout, tall, bearing numerous distinct thorns laterally or at tip, some bifurcated into two prongs with thorny tips	Chen et al. (2021a)
*Ophioplinthacagrenadensis* John and A. M. Clark, 1954	up to 5	separated oval proximally	up to 5 LOP, spiniform, 1 VMT	leaf-like, then slightly elongated with pointed tip along arm	DAP in first 1–2 ASE contiguous, then separated, VAP separated	flattened, covered by glassy spines	long, thick spinelets with rough thorny lateral surface in disc center, at periphery shorter	John and A. M. Clark (1954)
*Ophioplinthacahastata* Koehler, 1922	up to 7	contiguous, L = 1.5W	4–5 LOP, spiniform to club-shaped, distal ones largest, sometimes small granules present on distal edge of jaw, 1 VMT, 1 or 2 ASS	1^st^ pore 2, then 1, clavate, terminally spiniferous, longer than VAP	separated	smooth, DAS ≈ 3 × ASE length	numerous thorns at cylindrical tip	Koehler (1922), O’Hara and Stöhr (2006)
*Ophioplinthacaincisa* (Lyman, 1883)	up to 5	small, separated proximally, connected distally	4–5 LOP, spiniform., distal ones wide, flat, 1 VMT, 1 or 2 ASS	1^st^ pore 3, then 1	VAP separated	smooth, DAS ≈ 3 × ASE, VAS ≈ 1 × ASE length	cylindrical with thorny tip or thorny surface laterally, at disc periphery smooth	Lyman (1883)
*Ophioplinthacalaudator* Koehler, 1930	up to 7	small, separated proximally, connected distally	4 LOP, sometimes irregularly arranged, elongated, pointed but distalmost one flat and wide, 1 VMT	–	VAP separated beyond 2^nd^ASE, DAP separated	DAS thorny, VAS smooth, DAS ≈ 2 × ASE, VAS ≈ 1½ –2 × ASE length	cylindrical with 2–3 thorns at tip or lateral thorns, at disc periphery smooth, conical	Koehler (1930)
*Ophioplinthacalithosora* (H. L. Clark, 1911)	up to 6 or 7	long, narrow, separated	10–15 LOP including small granules at distal edge of jaw	1^st^ pore 3, 2^nd^ pore 2, then one, large, pointed tip	VAP separated, tetragonal	first 1–2 DAS, smooth, 3 × ASE length, with thorny tip, 3 thorny VAS	cylindrical, bifid or mostly with 3 thorns at tip or with lateral thorns	H. L. Clark (1911), this study
*Ophioplinthacamanillae* Guille, 1981	up to 6	as wide as long, contiguous	3 LOP, 1 VMT, rough edges, large, pointed, small granules at distal edge of jaw	oval, large, rough edges	DAP on first 1–2 ASE contiguous, then separated, VAP separated	DAS ≈ 3 × ASE, VAS ≈ 1 × ASE length, thorny	bifid or mostly with 3 divided thorns at tip, central spines longer than peripheral ones	Guille (1981)
*Ophioplinthacamiranda* Koehler, 1904	up to 5 or 6	separated proximally, connected distally	3 LOP, 1 large VMT	triangular	DAP and VAP contiguous	both DAS and VAS small, thorny, rugose and same length	cylindrical with thorny circular tip	Koehler (1904)
*Ophioplinthacamonitor* Koehler, 1930	up to 7 or 8	separated	4 LOP, 1 VMT, distalmost one smaller	1^st^ pore 2, then 1, oval to rounded proximally and pointed distally	DAP on first 2 ASE contiguous, then separated, fan-shaped	DAS with conspicuously sparse thorny surface, VAS thorny	granules with thorny tip	Koehler (1930), O’Hara and Stöhr (2006)
*Ophioplinthacapapillosa* H. L. Clark, 1939	up to 7	separated, narrow	3–4 LOP, narrow, subequal, long, pointed, 1 VMT	flat, moderately, large, pointed	separated	rough surface, DAS ≈ 3 × ASE, VAS ≈ 1 × ASE length	in disc center with long tip dividing into 2–3 thorns, at periphery with spinous tip	H. L. Clark (1939)
*Ophioplinthacaplicata* (Lyman, 1878)	up to 8	contiguous, L = 2–2.5W	3–5 LOP, 1–3 VMT, spiniform	1^st^ pore 2–3, curved inward, pointed round tip	DAP contiguous, VAP separated	thorny surface	conical, cylindrical, finely rugose or rarely with few longer thorns	Lyman (1878, 1882), Koehler (1904), O’Hara and Stöhr (2006)
*Ophioplinthacapulchra* Koehler, 1904	up to 7	separated proximally, connected distally	4 LOP, 1 VMT	leaf-like	first two VAP contiguous, then separated, DAP contiguous	thorny, rugose surface, uppermost DAS longest	cylindrical with thorny tip in center, at periphery smaller and conical	Koehler (1904, 1922), O’Hara and Stöhr (2006)
*Ophioplinthacarudis* (Koehler, 1897)	up to 5	completely separated or distally connected	5–6 LOP, spiniform, 1 VMT	leaf-like	DAP contiguous or separated, VAP widely separated	finely thorny	long spines with smooth surface	Koehler (1897), O’Hara and Stöhr (2006)
*Ophioplinthacasarsii* (Lyman, 1878)	up to 8	separated	3 LOP small, pointed, 1 VMT	flat, tapering, jagged	DAP, VAP separated	stout, cylindrical, glassy, blunt, very thorny	smooth cylindrical	Lyman (1878)
*Ophioplinthacasemele* (A.H. Clark, 1949)	up to 7	separated proximally, connected in distally, L ≈ 2½–3W	5 LOP, pointed, flatted, ill-defined, 1 VMT	1^st^ pore 3 or rarely 5, 2^nd^ pore 2–3, 3^rd^ pore 2, then one, large, pointed tip	VAP in first 1–2 ASE contiguous, then separated, DAP separated	long, slender, DAS with conspicuous thorns, VAS thorny surface	short, stout, with thorny tip, or 3 thorns, at periphery smooth spines without thorns	A. H. Clark (1949), Chen et al. (2021a), this study
*Ophioplinthacasexradia* Mortensen, 1933	up to 4	separated proximally, connected distally	3 LOP, 1 VMT	small, leaf-like	separated, small	small, thick base	irregular scales with conical tubercles	Mortensen (1933)
*Ophioplinthacaspinissima* H. L. Clark, 1900	up to 9	separated proximally, connected in distally, longer than wide	up to 5–7 LOP, spiniform, 1VMT	1^st^ pore one or divided into two, then more pointed distalwards along arm	DAP widely separated	thorny surface	spine with thorny tip, or 3 thorns, at periphery smooth spines without thorns, tip relatively flat with thorns	H. L. Clark (1900)
*Ophioplinthaca* sp.	up to 6	contiguous, distally convex, proximally triangular L = 2W	4–5 LOP, pointed, elongated, slightly flattened distal end, 1 VMT large, slightly longer than LOP	1^st^ pore 1–2, then 1, leaf-like, but along arm narrower, thorny, pointed; as long asVAP	both VAP and DAP widely separated	DAS has conspicuous lateral thorns; VAS with thorny surface	disc center: conical or short cylindrical spines, when cylindrical with two sub-thorns; at periphery smooth, conical or short, cylindrical, finely rugose to smooth	this study
*Ophioplinthacatylota* H. L. Clark, 1939	up to 6	separated proximally, connected distally	4 LOP, narrow, equal, long, pointed, 1 VMT	very thick, heavy, smooth but increasingly flatter along arm, smaller, very thorny	first and second DAP contiguous, then separated, VAP separated	thorny spine, DAS with conspicuous thorns, DAS ≈ 3 × ASE, VAS ≈ 1 × ASE length	smooth, rounded spine	H. L. Clark (1939)
*Ophioplinthacawebri* (Koehler, 1904)	up to 7	separated proximally, connected distally	4 LOP, narrow, equal, long, pointed, 1 large VMT	1^st^ pore 2–3, then 1, elongated	DAP and VAP contiguous	–	-	Koehler (1904)

Some species share morphological characters with the new species. *Ophioplinthacaglobata* Koehler, 1922 is similar to *O.brachispina* sp. nov. by having similar disc spine shape, arm spine shape, radial shields separated proximally and connected distally, number of lateral oral papillae, and separated ventral and dorsal arm plates, but differs by number of arm spines (up to six), the disc spines being scattered across the disc, radial shields separated by disc scales, characters of the oral shield, and a smooth surface on the arm plates along the entire arm. *Ophioplinthacahastata* Koehler, 1922 is similar to *O.brachispina* sp. nov. by having a slightly similar shape of the disc spines, separated dorsal and ventral arm plates, and similar tentacle scales on the distal end of the arm, but differs by number of arm spines (up to seven) and shape of dorsal arm spines, size of radial shields, characters of oral parts, and smooth arm surface. *Ophioplinthacaathena* A.H. Clark, 1949 is similar to *O.brachispina* sp. nov. by having similar disc spines with thorny tip, similar number of arm spines, separated radial shields, number of lateral oral papillae, but differs by large radial shields, thorny and leaf-like tentacle scales, separated dorsal and ventral arm plates. *Ophioplinthacaamezianeae* O’Hara & Stöhr, 2006 is similar to *O.brachispina* sp. nov. by having similar thorny tentacle scales, separated radial shields, separated dorsal and ventral arm plates, number of lateral oral papillae, but differs by number of arm spines, tall and thorny disc spines, and spiniform lateral oral papillae. *Ophioplinthacabythiaspis* (H. L. Clark, 1911) is similar to *O.brachispina* sp. nov. by having separated radial shields and number of lateral oral papillae, but differs by oval tentacle scales, conical disc spines, number of arm spines and contiguous dorsal arm plates. *Ophioplinthacagrenadensis* John & A. M. Clark, 1954 is similar to *O.brachispina* sp. nov. by having similar number of arm spines, separated radial shields, number of lateral oral papillae, and separated arm plates but differs by leaf-like thornless tentacle scales, long and thick disc spines. *Ophioplinthacaplicata* (Lyman, 1878) is similar to *O.brachispina* sp. nov. by having similar disc and arm spines, and number of lateral oral papillae, but differs by continues dorsal arm plates, pointed tentacle scale with rounded base, and contiguous radial shields. *Ophioplinthacarudis* (Koehler, 1897) is similar to *O.brachispina* sp. nov. by having similar thorny leaf-like tentacle scales, similar number of arm spines, separated radial shields, separated dorsal and ventral arm plates, but differs by number of lateral oral papillae, tall and thorny disc spines, and spiniform lateral oral papillae.

One of the most distinguishing characters to delimit the new species from almost all species in the genus *Ophioplinthaca* is the presence of spines with rough surface on lateral, ventral, and dorsal arm plates. The paratype (relatively smaller than the holotype) has thinner and denser spines on the arm. Although, some *Ophioplinthaca* species have a rough surface on dorsal arm plates or the distal margin covered with minute spines (*Ophioplinthacaplicata* and *Ophioplinthacaincisa*; (O’Hara 2010), this is the first record of a species with spines on the entire arm in the genus *Ophioplinthaca*.

##### ﻿ Ophioplinthaca

Taxon classificationAnimaliaAmphilepididaOphiacanthidae

sp.

55EFEFC4-751A-5C6F-B294-F0FE36784853

Figs 6 , 7

###### Material examined.

Northwest Pacific • 1 specimen; near Mariana Trench, Southwest of Guam Island, seamount; 12°36.44'N, 140°51.73'E; depth 2779 m; 23 September 2019; Collecting event: stn. SC038; Shenhaiyongshi msv leg; preserved in -80 °C; GenBank: OK043831; IDSSE-EEB-SW0108.

###### Description.

**Figure 6.**
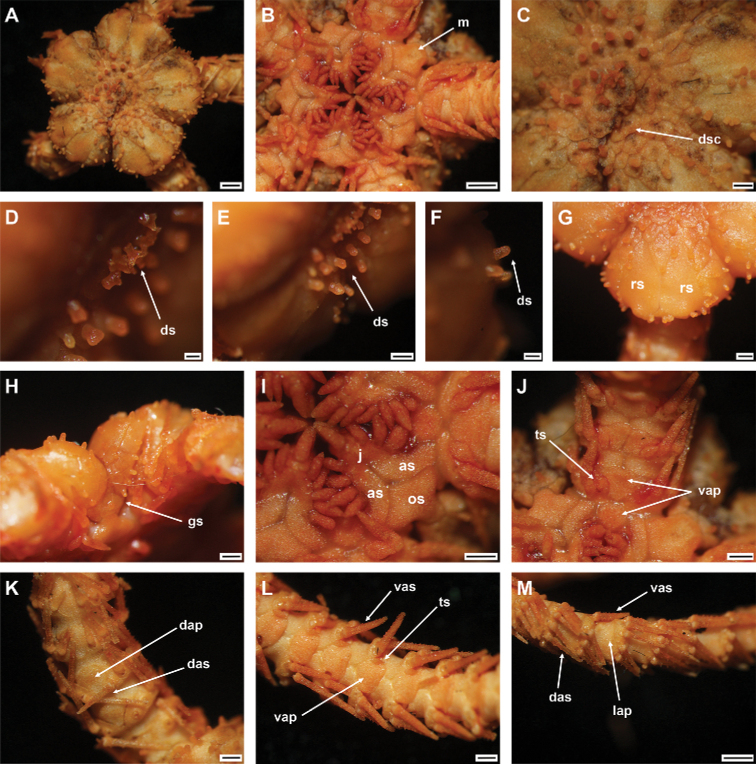
*Ophioplinthaca* sp. (IDSSE-EEB-SW0108) **A** dorsal disc **B** ventral disc **C** center of the disc **D–F** disc spines **G** radial shield **H** lateral disc **I** oral frame **J** ventral side of the arm base **K** dorsal arm **L** ventral arm **M** lateral arm. Abbreviations: **as** adoral shield, **dap** dorsal arm plate, **das** dorsal arm spine, **dp** disc plate, **gs** genital slit, **j** jaw, **lap** lateral arm plate, **m** madreporite, **os** oral shield, **rs** radial shield, **ts** tentacle scale, **vap** ventral arm plate, **vas** ventral arm spine. Scale bars: 1 mm (**A, B, M**); 500 µm (**C, E, G–L**); 200 µm (**D, F**).

Disc diameter 9 mm, arm base width 2 mm (Fig. 6).

***Disc*.** Disc sub-pentagonal, incised interradially to nearly 1/5 disc radius, creating five wedge-shaped lobes over each arm base in contrast to sunken center and interradii of disc (Fig. 6A, B). Disc scales polygonal to rounded, somewhat similar in size, overlapping at center (Fig. 6C). Most disc scales bear one or two spines (Fig. 6C). Disc spines at center 0.25–0.3 mm high, smooth, or finely rugose, cylindrical single base with two or three sub-thorns, which bend into opposite directions (Fig. 6D, E). Some disc spines at center 0.2–0.3 mm high, smooth, or finely rugose, cylindrical with large, blunt tip (Fig. 6D, E). Disc spines around radial shields and periphery of disc 0.2 mm high, smooth, or finely rugose cylindrical, with blunt rounded tip (Fig. 6E–G). Disc scales interradially slightly increasing in size distalwards, and between radial shields, with one to four spines (Fig. 6G, H). Radial shields naked, ~ ¼ disc diameter in length, 1.5–2 × as long as wide, with acute proximal end, and wide, slightly convex distal end (Fig. 6G). Radial shields connected, but at proximal end separated by disc scales, and surrounded by disc spines (Fig. 6G). Ventral disc covered by small disc scales similar to interradial dorsal scales, bearing spines similar to periphery of disc (Fig. 6H–J). Genital slits conspicuous and extending from oral shield to periphery of disc (Fig. 6H–J). Madreporite arrowhead-shaped, as wide as long, pentagonal with pointed proximal end, lobed distal edge with thickened lateral margins (Fig. 6B). Oral shields twice as wide as long, diamond-shaped with obtuse proximal end, concave lateral margins along the adoral shields, distal edge with central lobe (Fig. 6I). Adoral shield 3 × as long as wide, slightly curved, proximal edge concave, distal edge convex, but near first ventral arm plate straight, and pair of shields proximally connected (Fig. 6I). Adoral shields enclose proximal edges of oral shield, and partly separate oral shield from arm by connecting to lateral arm plate of first arm segment (Fig. 6I, J). Jaw large, triangular, longer than wide (Fig. 6I). One slightly pointed, and large ventralmost tooth, longer and thicker than the four to five long, spiniform lateral oral papillae (Fig. 6I). One round, scale-like small adoral shield spine located at lateral margin of adoral shield at edge of second tentacle pore, in some jaw angles (Fig. 6I).

***Arms*.** Five slightly moniliform arms, with smooth plates. Dorsal arm plates fan-shaped, as long as wide, widely separated, with convex distal edge, triangular proximal edge, straight lateral margins. Proximal edge of dorsal arm plate changes from obtuse to sharp triangular along arm (Fig. 6K). First ventral arm plate square to slightly trapezoid, as wide as long, with straight proximal and distal ends. Second and third ventral arm plate trapezoid, twice as wide as long, with straight proximal edge, slightly wavy distal edges, concave and diverging lateral edges (Fig. 6J). Second ventral arm plate contiguous with first ventral arm plate; following ventral arm plates as wide as long, pentagonal, with blunt to pointed proximal end, straight proximolateral margins, slightly curved lateral angles, straight to slightly curved inwards at distal end, and widely separated (Fig. 6J, L). Lateral arm plates meeting above and below (Fig. 6K–M). Up to six arm spines: three dorsal arm spines, two and a half arm segments in length, thorny or rarely smooth, lateral margins with scattered sharp thorns, apex pointed (Fig. 6M); three ventral arm spines, one to one and a half arm segments in length, pointed, and thorny or rough surface (Fig. 6K–M). First tentacle pore covered with one or two leaf-like, pointed tentacle scales (Fig. 6J). Following tentacle pores covered with one tentacle scale, as long as ventral arm plate, leaf-like, with thorny pointed tip (Fig. 6L).

***Color*.** In live specimen, pale orange-brown (Fig. 6).

**Figure 7.**
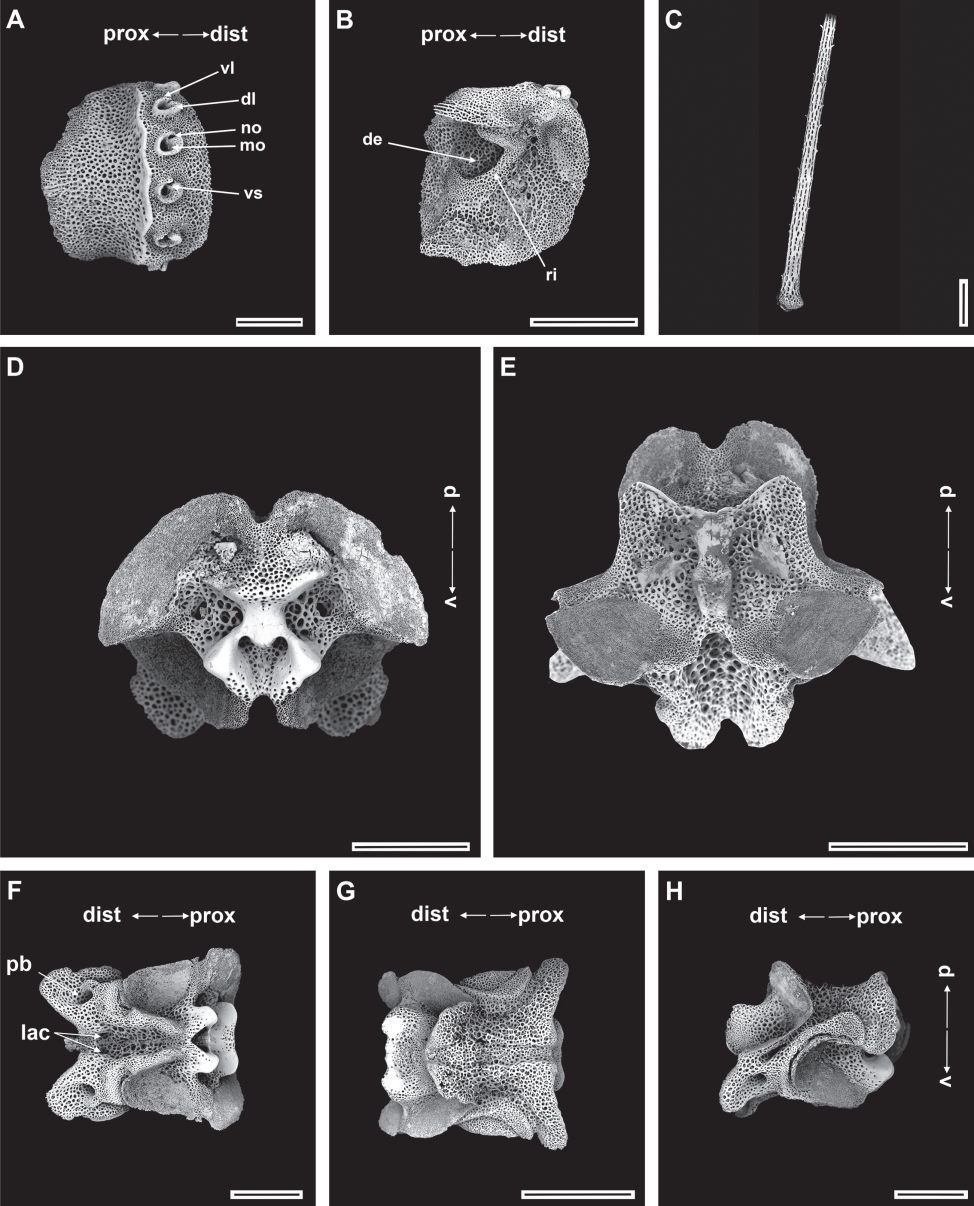
*Ophioplinthaca* sp. (IDSSE-EEB-SW0108) **A, B** lateral arm plate **C** dorsal arm spine **D–H** vertebrae **D** proximal view **E** distal view **F** ventral view **G** dorsal view **H** lateral view. Abbreviations: **d** dorsal, **de** depression, **dist** distal, **dl** dorsal lobe, **lac** lateral ambulacral canals, **mo** muscle opening, **no** nerve opening, **pb** podial basin, **prox** proximal, **ri** ridge, **th** thorns, **v** ventral, **vl** ventral lobe, **vs** volute-shape. Scale bars: 800 µm (**B**); 500 µm (**A, C–H**).

***Ossicle morphology*.** Arm spine articulations well developed, six in number, placed at slight angle to distal edge of lateral arm plate. A volute-shaped perforated lobe forms dorsal and distal parts of articulation (Fig. 7A). Arm spine articulation with large muscle opening and small nerve opening (Fig. 7A). Inner half of lateral arm plate with continuous ridge and prominent knob close to ventral edge forming vertebral articulation, shaped like a deep, nose-shaped beak (Fig. 7B). Dorsal arm spine thorny, with several longitudinal rows of perforations with widely spaced small thorns (Fig. 7C). Vertebrae with streptospondylous articulation, short, broad podial basin, and narrow small distal end (Fig. 7D–H). Dorsal end of vertebrae distally triangular and proximally flattened with longitudinal groove along midline (Fig. 7G). Ventral end of vertebrae with broad ambulacral groove, with lateral ambulacral canals (Fig. 7F, H).

###### Distribution.

2779 m depth, Northwest Pacific, near Mariana trench, Southwest of Guam Island.

###### Remarks.

*Ophioplinthaca* sp. shares morphological characters with many other *Ophioplinthaca* species, but can easily be delimited by the number of arm spines, disc spine shape, radial shields, and tentacle scale characters (Table 3). One of the distinguishing characters of this species is its smooth sub-thorny disc spines. According to the literature, only three *Ophioplinthaca* species have disc spines with sub-thorns on a single base (*Ophioplinthacaclothilde* A.H. Clark, 1949, *Ophioplinthacagrandisquama* Chen, Na & Zhang, 2021a, and *Ophioplinthacamanillae* Guille, 1981), but the disc spines are covered with numerous spinules in these species.

The most similar species to *Ophioplinthaca* sp. is *Ophioplinthacaclothilde* sharing contiguous radial shields, similar number of arm spines (up to seven), number and shape of lateral oral papillae, similar tentacle scale, and separated ventral and dorsal arm plates, but differs in longer dorsal arm spines (up to four arm segments), disc spines with single cylindrical base ending in two or three crowns, or a stout disc spine with irregular crown of a dozen or more spinules, more or less ovoid radial shields with convex proximal side, slightly contiguous dorsal arm plates on proximal arm segments, and equal size of ventralmost tooth and lateral oral papillae. We refrain from naming our specimen, as these differences may suggest an undescribed species or fall within the insufficiently known variability of *O.clothilde*. This question may be answered, when more specimens have been collected, and molecular data are needed for *O.clothilde*.

*Ophioplinthacaglobata* is similar to *Ophioplinthaca* sp. by having a similar number of arm spines (up to six), number of lateral oral papillae, and separated ventral and dorsal arm plate and shape, but differs by thorny disc spine and spine shape, separated radial shields and their shape, and tentacle scale longer than ventral arm plate. *Ophioplinthacalaudator* Koehler, 1930 shares with *Ophioplinthaca* sp. almost the same number of arm spines (up to seven), by size of radial shields, number and shape of lateral oral papillae, and separated dorsal and ventral arm plates, but differs in thorny disc spines, with two to three thorns or sub-thorns on their lateral surface, separated radial shields, and smooth arm spines. *Ophioplinthacagrandisquama* is similar to *Ophioplinthaca* sp. by having contiguous radial shields, closer number of arm spines (up to seven), and by the shape of arm and disc spines, but differs by tall (0.8 mm in high) long, thorny disc spines with two or three thorny sub-crowns, blunt tentacle scale, and contiguous ventral and dorsal arm plates. *Ophioplinthacamanillae* Guille, 1981 is similar to *Ophioplinthaca* sp. by having similar number and shape of arm spines, contiguous radial shields, shape of lateral oral papillae, and separated dorsal and ventral arm plates, but differs in an oval tentacle scale, and in height and shape of disc spines.

##### ﻿ Ophioplinthaca amezianeae

Taxon classificationAnimaliaAmphilepididaOphiacanthidae

O’Hara & Stöhr, 2006

DB59B789-34D2-546B-84B2-A49EE47453C4

Figs 8 , 9

Ophioplinthaca amezianeae O’Hara & Stöhr, 2006: 77–78, fig. 9D–G.

###### Material examined.

Northwest Pacific • 1 specimen; near Mariana Trench, Southwest of Guam Island, seamount; 11°40.33'N, 141°20.57'E; depth 3600 m; 27 November 2020; Collecting event: stn. SC040; Shenhaiyongshi msv leg; preserved in -80 °C; GenBank: OK043832; IDSSE-EEB-SW0109.

###### Description.

**Figure 8.**
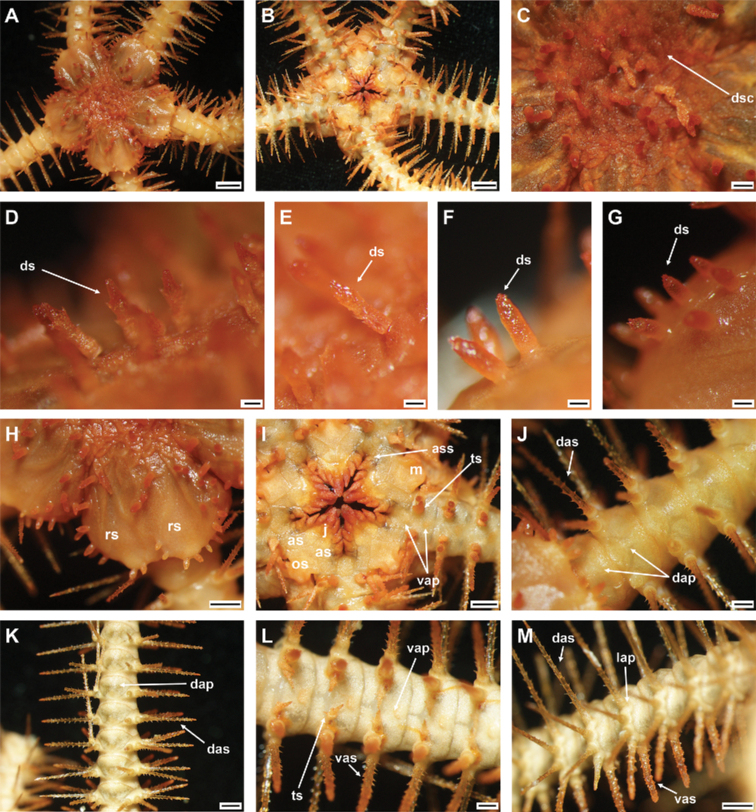
*Ophioplinthacaamezianeae* O’Hara & Stöhr, 2006 (IDSSE-EEB-SW0109) **A** dorsal disc **B** ventral disc **C** center of the disc **D–G** disc spines **H** radial shield **I** oral frame **J** dorsal side of the arm base **K** dorsal arm **L** ventral arm, **M** lateral arm. Abbreviations: **as** adoral shield, **ass** adoral shield spine, **dap** dorsal arm plate, **das** dorsal arm spine, **dp** disc plate, **gs** genital slit, **j** jaw, **lap** lateral arm plate, **m** madreporite, **os** oral shield, **rs** radial shield, **ts** tentacle scale, **vap** ventral arm plate, **vas** ventral arm spine. Scale bars: 2 mm (**A, B**); 1 mm (**H, I, K, M**); 500 µm (**C, J, L**); 200 µm (**D–G**).

Disc diameter 11.5 mm, arm base width 2.5 mm (Fig. 8).

***Disc*.** Sub-pentagonal and incised interradially to 1/8 disc radius, creating five wedge-shaped lobes over each arm base in contrast to sunken center and interradii of disc (Fig. 8A, B). Disc scales variable in size, overlapping, dense at center, and some scales bear spines (Fig. 8C). Scales increase in size distalwards from disc center to distal end of radial shields interradially (Fig. 8C–G). Disc spines at disc center 0.7–0.9 mm high, thick, with cylindrical to rounded base, tapering to a sharp point, or terminating in usually one or two small thorns, with additional irregular flanged thorns arising from lateral margins along the spine (Fig. 8D, E). Disc spines on disc periphery and around radial shields, slightly smaller than center spines (0.5–0.7 mm in height), cylindrical, finely rugose, with thorny blunt tip (Fig. 8F, G). Radial shields large, twice as long as wide, with acute proximal end, much wider convex distal end, and completely separated by disc scales (Fig. 8H). Ventral disc covered by smaller scales compared to dorsal scales and overlapped, without or rarely bearing spines (Fig. 8B, I). Genital slits conspicuous and extending from oral shield to periphery of disc (Fig. 8I). Madreporite arrowhead-shaped, as wide as long, triangular with pointed proximal end, convex distal edge with thickened lateral margins (Fig. 8I). Oral shield arrowhead-shaped, as wide as long, triangular with pointed proximal end, slightly concave lateral margins along adoral shields, lobed distal edge with rounded lateral margins (Fig. 8I). Adoral shield 3 × as long as wide, with straight lateral margin, and pair of shields proximally connected (Fig. 8I). Jaw longer than wide, and oral plates concealed by adoral shields (Fig. 8I). Jaw bearing one large, pointed ventralmost tooth with three pointed, rod-like lateral oral papillae, shorter than ventralmost tooth, finely rugose, with wide, rounded base, and pointed tip (Fig. 8I). One small, oval adoral shield spine at lateral margin of adoral shield at edge of second tentacle pore (Fig. 8I).

***Arms*.** Five slightly moniliform arms, with smooth plates. Dorsal arm plates fan- to bell-shaped, with truncated proximal end on first dorsal arm plate, but following plates with obtuse proximal end, straight to slightly convex proximolateral margins, and convex distal margin (Fig. 8J, K). Dorsal arm plates at proximal end of arm barely separated, but distally widely separated (Fig. 8J, K). First ventral arm plate trapezoid, as wide as long, with sunken proximal end, distally connected to second ventral arm plate (Fig. 8I). Following ventral arm plates twice as wide as long, with obtuse proximal end, straight proximolateral margins, straight lateral angles, straight to slightly wavy distal end, and widely separated (Fig. 8L). Lateral arm plates meeting above and below. Up to five arm spines: two dorsal spines, three arm segments in length, slender, thorny, laterally compressed with row of tall sharp thorns (Fig. 8J–M); two ventral spines, two arm segments in length, thick, with blunt tip, rugose, and thorny surface (Fig. 8J, K). First tentacle pore covered by one or two tentacle scales with rounded base and pointed tip (Fig. 8I). Following tentacle scales with rounded base, spiniform, pointed tip and covered in irregular thorns, mostly on middle to distal half of arm (Fig. 8L).

***Color*.** In live specimen, orange-brown dorsal disc, pale color on arms and ventral disc, arm spines orange, and disc spines red (Fig. 8).

**Figure 9.**
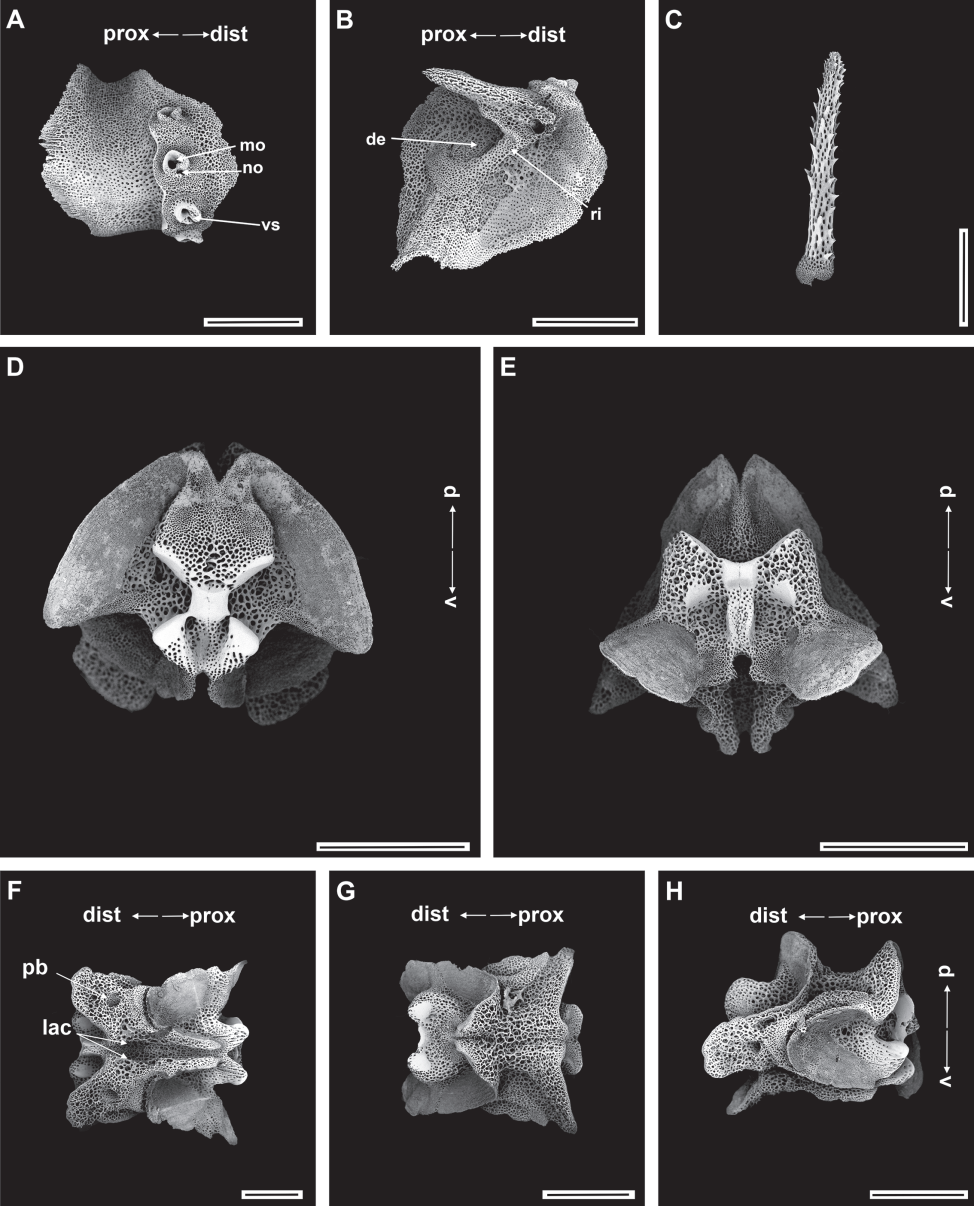
*Ophioplinthacaamezianeae* O’Hara & Stöhr, 2006 (IDSSE-EEB-SW0109) **A, B** lateral arm plate **C** ventral arm spine **D–H** vertebrae **D** proximal view **E** distal view **F** ventral view **G** dorsal view **H** lateral view. Abbreviations: **d** dorsal, **de** depression, dist distal, **lac** lateral ambulacral canals, **mo** muscle opening, **no** nerve opening, **pb** podial basin, **prox** proximal, **ri** ridge, **th** thorns, **v** ventral, **vs** volute-shape. Scale bars: 800 µm (**A–E, G, H**); 500 µm (**F**).

***Ossicle morphology*.** Arm spine articulations well developed, four in number, and placed at slight angle to distal edge of lateral arm plate. Volute-shaped perforated lobe forms dorsal and distal part of articulation (Fig. 9A); large muscle opening, small nerve opening (Fig. 9A). Proximal half of lateral arm plate internal surface with continuous ridge and prominent knob close to ventral edge forming vertebral articulation, shaped like a broad, nose-shaped beak (Fig. 9B). Ventral arm spine thorny, with blunt apex, several longitudinal rows of perforations and small thorns (Fig. 9C). Vertebrae with streptospondylous articulation, short, broad podial basin at proximal end, and narrow small distal end (Fig. 9D–H). Dorsal end of vertebrae distally triangular and proximally flattened with longitudinal groove along midline (Fig. 9D, E). Ventral end of vertebrae with broad ambulacral groove with lateral ambulacral canals (Fig. 9F–H).

###### Distribution.

1618–3600 m depth, Southwest of Guam Island, Northwest Pacific, New Caledonia, New Zealand.

###### Remarks.

*Ophioplinthacaamezianeae* was described by O’Hara and Stöhr (2006), and recorded from deep waters in the South Pacific region. It can easily be delimited from most species in the genus *Ophioplinthaca* by disc spine, radial shield, and tentacle scale characters (Table 3).

*Ophioplinthacaamezianeae* from the present study is similar to the holotype description, but it differs slightly in the disc spine shape and number of arm spines on the lateral arm plate. However, the number of arm spines differs between individuals (6–10 arm spines) according to the description of paratype variations of *O.amezianeae* (O’Hara and Stöhr 2006). The disc spines of our specimen are slightly thicker than in the holotype, but their shape and irregular flanged thorns arising from lateral margins along the spine are similar to the holotype description. The holotype is significantly larger than our specimen (14.5 mm disc diameter), and *Ophioplinthaca* species usually show high intraspecific morphological variation. Therefore, a slight difference in disc spine thickness can be considered as intraspecific morphological variation within *O.amezianeae*.

##### ﻿ Ophioplinthaca athena

Taxon classificationAnimaliaAmphilepididaOphiacanthidae

A. H Clark, 1949

94024E65-5460-5217-89FC-5D010B3EA05F

Figs 10 , 11

Ophioplinthaca athena A. H Clark, 1949: 23–24, fig. 9; Chen et al. 2021b: 60–61, fig. 3.

###### Material examined.

Northwest Pacific • 1 specimen; near Mariana Trench, Southwest of Guam Island, seamount; 12°8.83'N, 139°0.37'E; depth 1987 m; 27 November 2020; Collecting event: stn. SC041; Shenhaiyongshi msv leg; preserved in -80 °C; GenBank: OK043833; IDSSE-EEB-SW0110.

###### Description.

Disc diameter 12.5 mm, arm base width 1.5 mm (Fig. 9).

**Figure 10.**
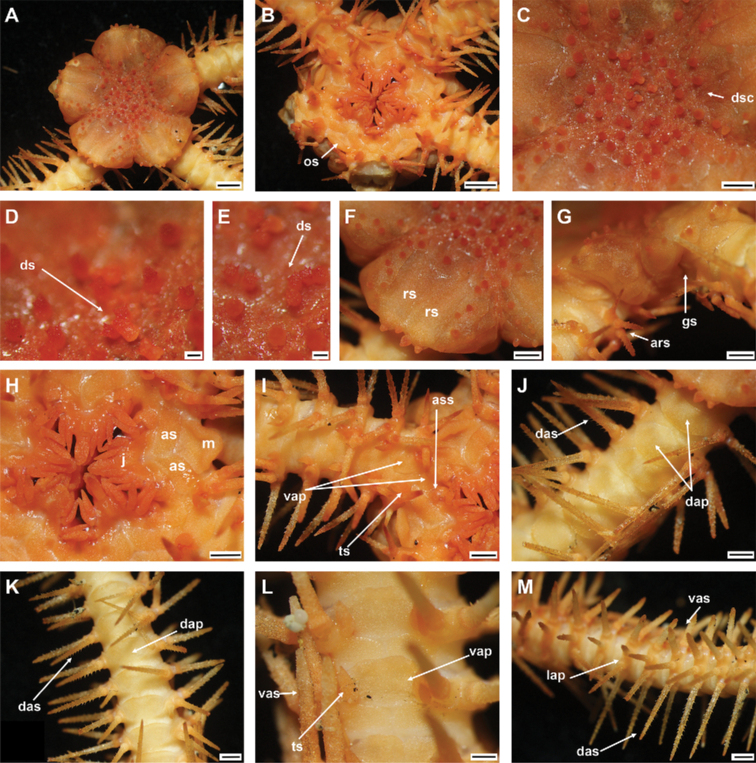
*Ophioplinthacaathena* A. H. Clark, 1949 (IDSSE-EEB-SW0110) **A** dorsal disc **B** ventral disc **C** center of the disc **D–E** disc spines **F** radial shield **G** lateral disc **H** оral frame **I** ventral side of the arm base **J** dorsal side of the arm base **K** dorsal arm **L** ventral arm **M** lateral arm. Abbreviations: **as** adoral shield, **ass** adoral shield spine, **dap** dorsal arm plate, **das** dorsal arm spine, **dp** disc plate, **gs** genital slit, **j** jaw, **lap** lateral arm plate, **m** madreporite, **os** oral shield, **rs** radial shield, **ts** tentacle scale, **vap** ventral arm plate, **vas** ventral arm spine. Scale bars: 2 mm (**A, B**); 1 mm (**C, F–K, M**); 500 µm (**L**); 200 µm (**D, E**).

***Disc*.** Sub-circular and incised interradially, creating five wedge-shaped lobes over each arm base in contrast to sunken center and interradii of disc (Fig. 10A, B). Disc scales small, irregular, overlapping, and some scales bear more than one stump (Fig. 10C–F). Scales increase in size distalwards from disc center to periphery interradially (Fig. 10D–F). Disc stumps in disc center with cylindrical base and few radiating spinules at truncated tip (Fig. 10D–F). Spines at disc periphery and around radial shields, slightly smaller, less cylindrical, more conical, smooth, with pointed tip (Fig. 10D, E). Radial shields large, 3 × as long as wide, acute proximal end, much wider and slightly convex distal end, pairs separated along proximal half, and barely connected distally (Fig. 10F). Ventral disc covered by small, overlapping disc scales without or rarely bearing conical granules (Fig. 10B, G). Genital slits conspicuous and extending from oral shield to periphery of disc (Fig. 10G). Madreporite arrowhead-shaped, as wide as long, triangular with pointed proximal end, lobed distal edge with thickened lateral margins. Other oral shields widely triangular, twice as wide as long, wide proximal angle, distal edge folded ventralwards with minute central lobe, and lateral angle connected to first lateral arm plate (Fig. 10H). Adoral shield 2 × as long as wide, with concave proximolateral margin, pair of shields proximally connected, and connected to first lateral and ventral arm plates (Fig. 10H). Jaw longer than wide, bearing one slightly blunt, flat, elongated, and large ventralmost tooth and four elongated, spiniform lateral oral papillae (Fig. 10H). Lateral oral papillae, finely rugose, equal in height to ventralmost tooth, with pointed tip (Fig. 10H). One small scale-like rounded adoral shield spine at lateral margin of adoral shield at edge of second tentacle pore (Fig. 10I).

***Arms*.** Five slightly moniliform arms, with smooth plates. Dorsal arm plates twice as long as wide, with truncated proximal end in first dorsal arm plate (Fig. 10J), but following plates with triangular proximal end, slightly curved proximolateral margins, and convex to slightly wavy distal margins covered with minute spines (Fig. 10J, K). Dorsal arm plates at proximal to middle arm segments barely separated, but distally widely separated (Fig. 10J, K). First ventral arm plate trapezoid, as wide as long, with sunken proximal end, and distal end connected to second ventral arm plate (Fig. 10I). Following ventral arm plates twice as wide as long, with obtuse proximally, straight proximolateral margins, curved lateral angles, straight to slightly wavy distal end, distal margins covered with minute spines, and widely separated (Fig. 10L). Lateral arm plates meeting above and below (Fig. 10K–M). Up to five arm spines. Proximal arm segment bearing two dorsal and three ventral arm spines (Fig. 10M). Dorsalmost arm spines at proximal end two to two and a half arm segments in length, smooth or with few thorns at lateral edge (Fig. 10M). Next dorsal arm spine much longer, nearly four arm segments in length, smooth or with thorns at lateral margin (Fig. 10M). Ventral arm spines short, less conspicuous thorns, more rugose surface (Fig. 10L, M). First tentacle pore covered with two leaf-like tentacle scales with pointed tip (Fig. 10H, I). Following pores covered with leaf-like pointed tentacle scale with rounded base and tip covered in micro spinules (Fig. 10L).

***Color*.** In live specimen, orange-brown dorsal disc, light color in arms and ventral disc, arm spines orange, disc spines and papillae red (Fig. 10).

**Figure 11.**
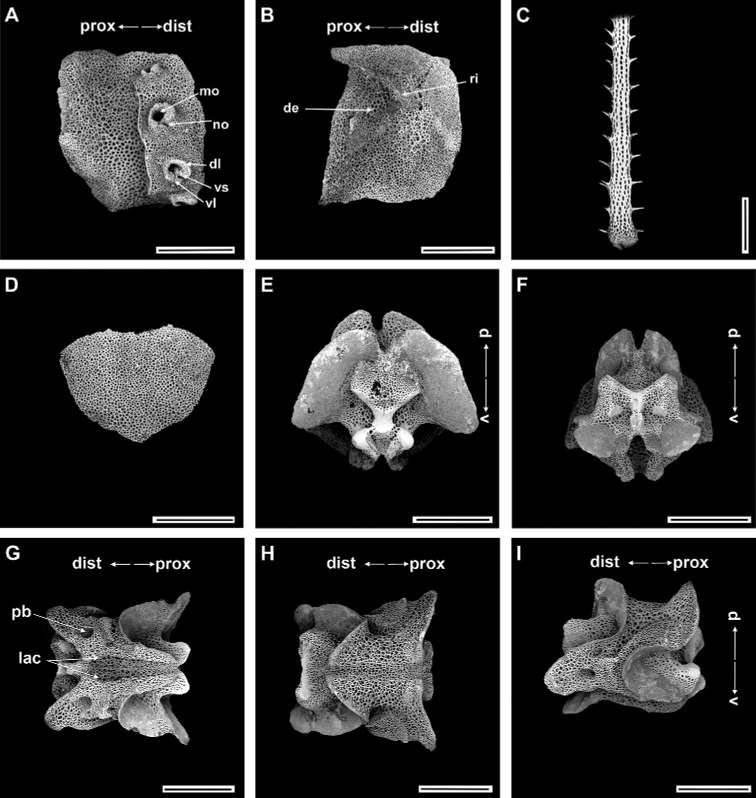
*Ophioplinthacaathena* A. H. Clark, 1949 (IDSSE-EEB-SW0110) **A, B** lateral arm plate **C** dorsal arm spine **D** dorsal arm plate **E–I** vertebrae **E** proximal view **F** distal view **G** ventral view **H** dorsal view **I** lateral view. Abbreviations: **d** dorsal, **de** depression, **dist** distal, **dl** dorsal lobe, **lac** lateral ambulacral canals, **mo** muscle opening, **no** nerve opening, **pb** podial basin, **prox** proximal, **ri** ridge, **th** thorns, **v** ventral, **vl** ventral lobe, **vs** volute-shape. Scale bars: 800 µm (**A–I**).

***Ossicle morphology*.** Arm spine articulations well developed, four in number, and placed at slight angle to distal edge of lateral arm plate. Volute-shaped perforated lobe forms dorsal and distal part of articulation, with large muscle opening and small nerve opening (Fig. 11A). Distal half of inner side of lateral arm plate with group of small, irregular perforations parallel to row of spine articulations; a continuous ridge and a prominent knob close to ventral edge form vertebral articulation, shaped like a broad, nose-shaped beak (Fig. 11B). Dorsal arm spine thorny, with several longitudinal rows of perforations and widely spaced tall thorns (Fig. 11C). Dorsal arm plate triangular with smooth surface (Fig. 11D). Vertebrae with streptospondylous articulation, short, broad podial basin at proximal end and narrow small distal end (Fig. 11E–I). Dorsal end of vertebrae distally triangular and proximally flattened with longitudinal groove along midline (Fig. 11E, F). Ventral end of vertebrae with broad ambulacral groove and lateral ambulacral canals (Fig. 11G–I).

###### Distribution.

1866–2157 m depth, Southwest of Guam Island, Northwest Pacific, Kupuai, Hawaii Islands.

###### Remarks.

*Ophioplinthacaathena* was described by A. H Clark (1949), and recorded from deep waters in the Hawaiian Islands. *Ophioplinthacaathena* resembles *O.papillosa*, *O.globata*, *O.hastata*, *O.plicata*, *O.carduus*, *O.semele*, *O.clothilde*, and *O.dipsacos* in disc spine characters, but differs in arm spine, oral frame, and radial shield characters (Table 3).

*Ophioplinthacaathena* from the present study is similar to the holotype description, but it differs slightly by separated dorsal arm plates and the shape of the dorsal arm spines, although the latter varies within our individual. Therefore, the shape of the arm spines is not a suitable morphological character to delimit *O.athena*. The description of the holotype mentioned that dorsal arm plates were contiguous, but in our specimen, they are just separated along the arm, and there are no paratypes of *O.athena*. Therefore, this difference may be related to the size of the specimen (holotype 14.5 mm disc diameter; A. H. Clark 1949), and these small morphological differences can be considered as intraspecific variation within *O.athena*.

##### ﻿ Ophioplinthaca cf. lithosora

Taxon classificationAnimaliaAmphilepididaOphiacanthidae

(H. L. Clark, 1911)

E9775B4E-4139-588A-8B92-7FB53323798B

Figs 12 , 13

Ophiocamax lithosora H. L. Clark, 1911: 191–193, fig. 89.
Ophiomitra lithosora : Matsumoto 1917: 131.

###### Material examined.

China • 1 specimen; South China Sea, near Xisha Islands archipelago, seamount; 16°47.79'N, 113°15.04'E; depth 602 m; 31 March 2020; Collecting event: stn. SC009; Shenhaiyongshi msv leg; preserved in-80 °C; GenBank: OK043834IDSSE-EEB-SW0111.

###### Comparative material.

Japan • ***Holotype*** specimen; East China Sea, Osumi Islands, Kuchnioerabu Island; 30°22'N, 129°08.5'E; depth 660 m; 13 Aug 1906; Collecting event: stn. 4918; R/V Abatross, North Pacific Expedition leg; preserved dry; USNM 25622. • 1 ***paratype*** specimen; North Pacific Ocean, Wakayama, Honshu Island, Shiono Misaki; 33°25.17'N, 135°37.33'E; depth 446–463 m; 29 Aug 1906; Collecting event: stn. 4967; R/V Abatross, North Pacific Expedition leg.; preserved dry; USNM 26220. • 1 specimen; S off Daiozaki, Kumanonada; 34°05.12'N, 136°51.24'E to 34°05.05'N, 136°50.5'E; depth 475–494 m; 25 May 1994; Collecting event: stn. KN25; R/V Tansei-Maru KT-94-07 leg.; gear 3 m ORE beam trawl; preserved in ethanol; NSMT E-7943.

###### Description.

**Figure 12.**
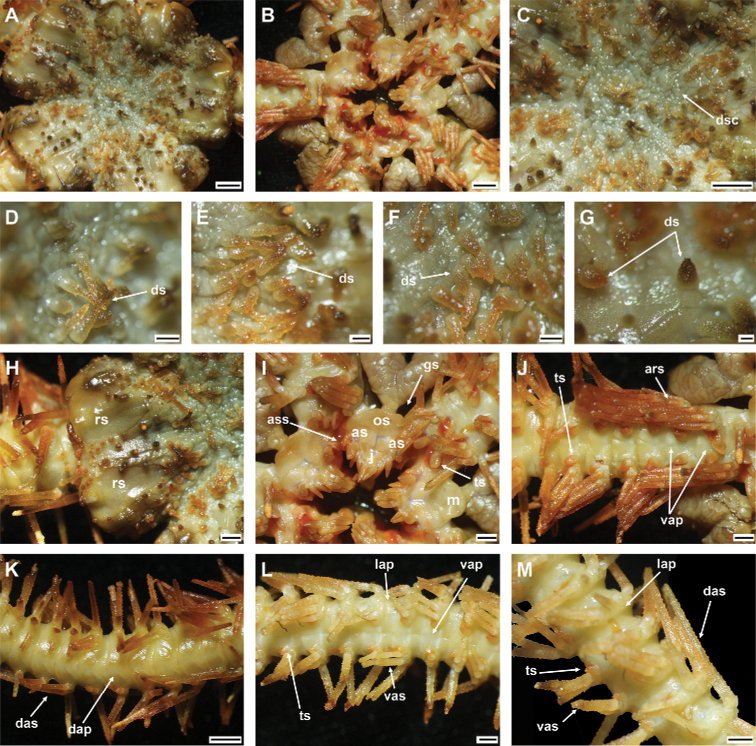
Ophioplinthacacf.lithosora (H. L. Clark, 1911) (IDSSE-EEB-SW0111) **A** dorsal disc **B** ventral disc **C** center of the disc **D–G** disc spines **H** radial shield **I** оral frame **J** ventral side of the arm base **K** dorsal arm **L** ventral arm, **M** lateral arm. Abbreviations: **ars** arm spine, **as** adoral shield, **ass** adoral shield spine, **dap** dorsal arm plate, **das** dorsal arm spine, **dp** disc plate, **gs** genital slit, **j** jaw, **lap** lateral arm plate, **m** madreporite, **os** oral shield, **rs** radial shield, **ts** tentacle scale, **vap** ventral arm plate, **vas** ventral arm spine. Scale bars: 2 mm (**A–C, K**); 1 mm (**H–J, L, M**); 500 µm (**D, E**); 200 µm (**F, G**).

Disc diameter 20 mm, arm base width 4.5 mm (Fig. 12).

***Disc*.** Sub-pentagonal and incised interradially, creating five wedge-shaped lobes over each arm base in contrast to sunken center and interradii of disc (Fig. 12A, B). Disc scales small, irregular, overlapping, some scales bear more than one low stout spine, these spread across entire disc except radial shields (Fig. 12C). Spines in disc center, 0.9–1.4 mm high, thick, with cylindrical to rounded base, tapering to sharp point, with truncated or pointed tip, and additional irregular flanged thorns arising from lateral margins along spine. Proximal end of disc spines, 0.8–0.9 mm high, thick, base cylindrical, with thorny pointed tip (Fig. 12D–G). Peripheral disc spines decreasing in size, conical, with smooth or slightly thorny pointed tip (Fig. 12F, G). Radial shields large, 2 × as long as wide, acute proximal end, much wider convex distal end, completely separated (Fig. 12H). Ventral disc covered by small, overlapping disc scales without spines (Fig. 12B, I). Genital slits conspicuous and extending from oral shield to periphery of disc (Fig. 12B, I). Oral shield wide fan-shaped, 2 × as wide as long, with rounded proximal end, concave lateral margins, convex to wavy distal edge, and lateral angle connected to first lateral arm plate (Fig. 12I). Madreporite as long as wide, proximal end with wide angle. Distal edge strongly convex (Fig. 12I). Adoral shield 2 × as long as wide, with straight lateral margin, pair of shields proximally connected (Fig. 12I). Adoral shields connected to first lateral and ventral arm plates (Fig. 12I). Jaw longer than wide, bearing four to five elongated, pointed leaf-like lateral oral papillae, cluster of up to three pointed tooth papillae, and small, 4–6 granules covered by adoral shield spines in some jaw slits (Fig. 12I). Two adoral shield spines, one larger, oval, scale-like flat, other one smaller, situated at lateral margin of adoral shield at second tentacle pore (Fig. 12I).

***Arms*.** Dorsal arm plates pentagonal, wider than long, with truncated proximal end, weakly convex proximolateral margins, straight lateral margins, and convex to slightly wavy distal margins, on proximal to middle arm segments contiguous, but distally separated (Fig. 12K). First ventral arm plate nearly square, connected to second ventral arm plate (Fig. 12J). Following ventral arm plates twice as wide as long, with obtuse proximal end, straight proximolateral margins, slightly concave lateral edges, straight distal edge, and widely separated (Fig. 12L). Lateral arm plates meeting only below (Fig. 12K–M). Up to seven arm spines, three dorsal and four ventral; dorsal arm spines two to three arm segments in length, thick, with smooth or rugose surface and lateral thorns; ventral arm spines shorter, two arm segments in length, smooth, or slightly rugose, thick, with pointed tip (Fig. 12L). First tentacle pore covered by two or three thickened, oval, blunt tentacle scales (Fig. 12J). Subsequent seven to eight tentacle pores covered by single similar oval scale (Fig. 12J). Further tentacle pores covered by one small scale, with rounded base and spinules at tip (Fig. 12L).

***Color*.** In live specimen, orange-brown dorsal disc, pale color in arms and ventral disc, arm spines orange, disc spines and papillae red (Fig. 12).

**Figure 13.**
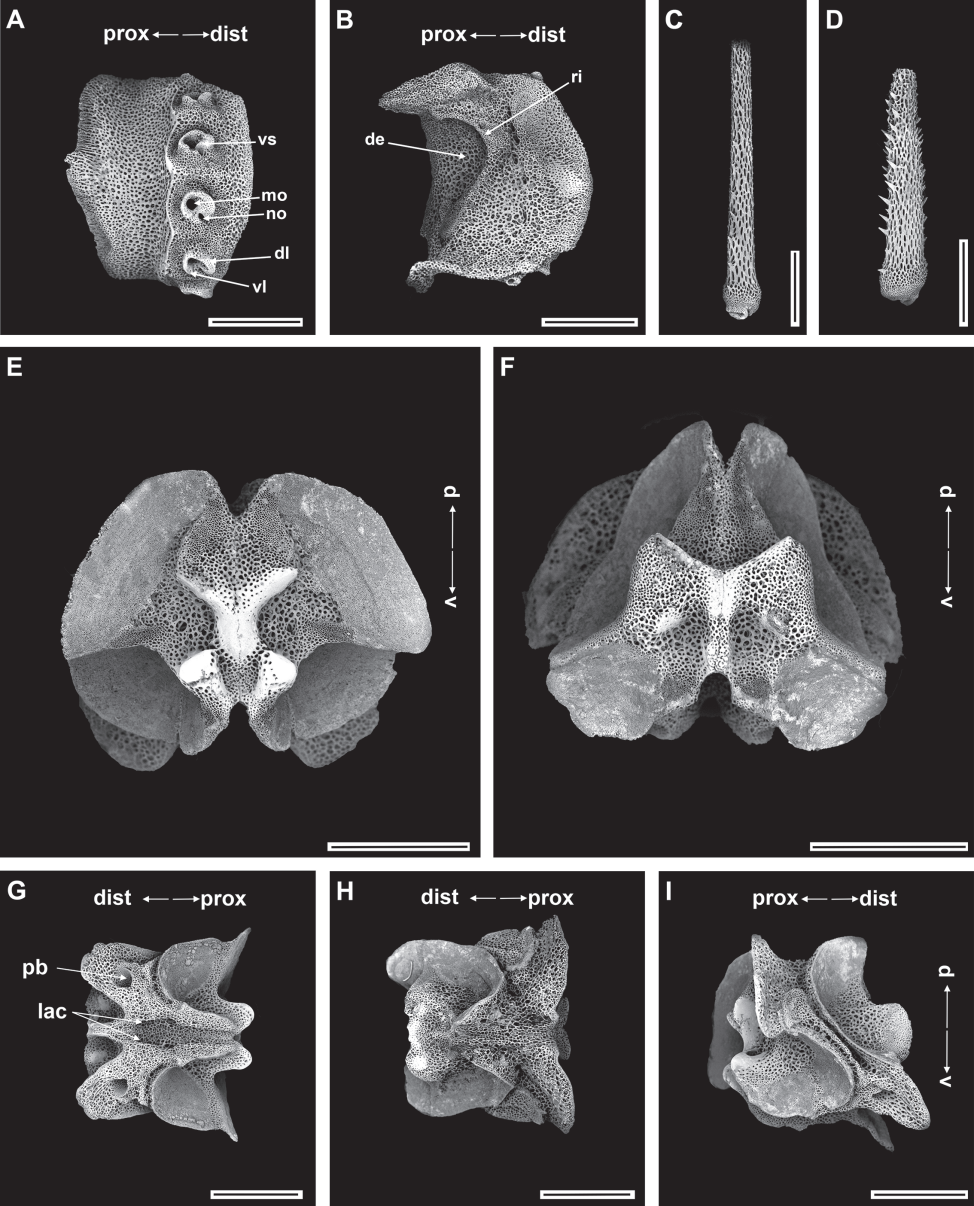
Ophioplinthacacf.lithosora (H. L. Clark, 1911) (IDSSE-EEB-SW0111) **A, B** lateral arm plate **C** dorsal arm spine **D** ventral arm spine **E–I** vertebrae **E** proximal view **F** distal view **G** ventral view **H** dorsal view **I** lateral view. Abbreviations: **d** dorsal, **de** depression, **dist** distal, **dl** dorsal lobe, **lac** lateral ambulacral canals, **mo** muscle opening, **no** nerve opening, **pb** podial basin, **prox** proximal, **ri** ridge, **th** thorns, **v** ventral, **vl** ventral lobe. Scale bars: 800 µm (**A–I**).

***Ossicle morphology*.** Arm spine articulations well developed, five in number, and placed at slight angle to distal edge of lateral arm plate. Volute-shaped perforated lobe forms dorsal and distal part of articulation, with large muscle opening and small nerve opening, and decreasing in size ventralwards (Fig. 13A). Distal half of inner side of lateral arm plate with group of small, irregular perforations parallel to row of spine articulations; a continuous ridge and a prominent knob close to ventral edge form vertebral articulation, shaped like a broad, nose-shaped beak (Fig. 13B). Dorsal arm spine thorny, with several longitudinal rows of perforations and widely spaced small thorns (Fig. 13C). Ventral arm spine short, rough, thorny surface with truncated apex (Fig. 13D). Vertebrae with streptospondylous articulation, short, broad podial basin at proximal end and narrow small distal end (Fig. 13E–I). Dorsal end of vertebrae distally acute and proximally flattened with longitudinal groove along midline (Fig. 13E, F). Ventral end of vertebrae with broad ambulacral groove and lateral ambulacral canals (Fig. 13G–I).

###### Distribution.

462–663 m depth, South China Sea, East China Sea, Japan Sea.

###### Remarks.

*Ophioplinthacalithosora* was described by H. L. Clark (1911) in the genus *Ophiocamax* Lyman, 1878, and is currently accepted in *Ophioplinthaca* (Stöhr et al. 2021). However, we could not find the taxonomic act that transferred it to *Ophioplinthaca* and assume that this decision may never have been formalized in a publication. We agree that it belongs in this genus on account of its deeply incised disc. Matsumoto (1917) considered *O.lithosora* in *Ophiomitra* Lyman, 1869, despite noticing the incised disc and enlarged marginal disc scales. These genera and *Ophiocamax* share indeed many other characters, but by molecular data they have not been found to be closely related and at least *Ophiomitra* may be polyphyletic (Christodoulou et al. 2019). *Ophioplinthacalithosora* was previously recorded from the East China Sea and Japan Sea. It is easily recognized within the genus *Ophioplinthaca* by radial shield, oral frame, and tentacle scale characters (Table 3).

Ophioplinthacacf.lithosora from the present study concurs with the holotype description in most respects, but it differs slightly by having contiguous dorsal arm plates, long spines in the center of the disc and few granules in the mouth angle at only some jaws. These granules are present at the second tentacle pore at the adoral shield at all jaw angles in the holotype, paratype and an additional specimen, all of which we examined from digital photographs. They are more obvious than in any other species of *Ophioplinthaca* and H. L. Clark (1911) included them in the series of oral papillae, which explains his high number of up to 15 lateral papillae at a single jaw edge. They may perhaps form a funnel around the tube foot. None of these specimens has long disc spines. However, the information about morphological variations within *O.lithosora* is still incomplete (H. L. Clark 1911), because it appears to be a rare species of which only few specimens are known. Therefore, these small morphological changes may represent intraspecific variation between *O.lithosora* specimens. We still cautiously refrain from fully associating our specimen with this species, due to the small differences between species in *Ophioplinthaca*.

##### ﻿ Ophioplinthaca semele

Taxon classificationAnimaliaAmphilepididaOphiacanthidae

(A. H Clark, 1949)

818A238B-B677-5083-8895-050FC5C469A9

Fig. 14

Ophiomitra semele A. H Clark, 1949: 20–23, fig. 8a, b.
Ophioplinthaca semele : O’Hara and Stöhr 2006: 76; Chen et al. 2021a: 14–18, fig. 6–8.

###### Material examined.

Northwest Pacific • 1 specimen; near Mariana Trench, Southeast of Guam Island, seamount, 12°6.67'N, 141°37.27'E; depth 1160 m; 03 September 2019; Collecting event: stn. SC033; Shenhaiyongshi msv leg; preserved in -80 °C; GenBank: OK043835, IDSSE-EEB-SW0113.

###### Distribution.

537–1987 m depth, southwest of Guam Island, Northwest Pacific, Hawaii Islands.

###### Remarks.

**Figure 14.**
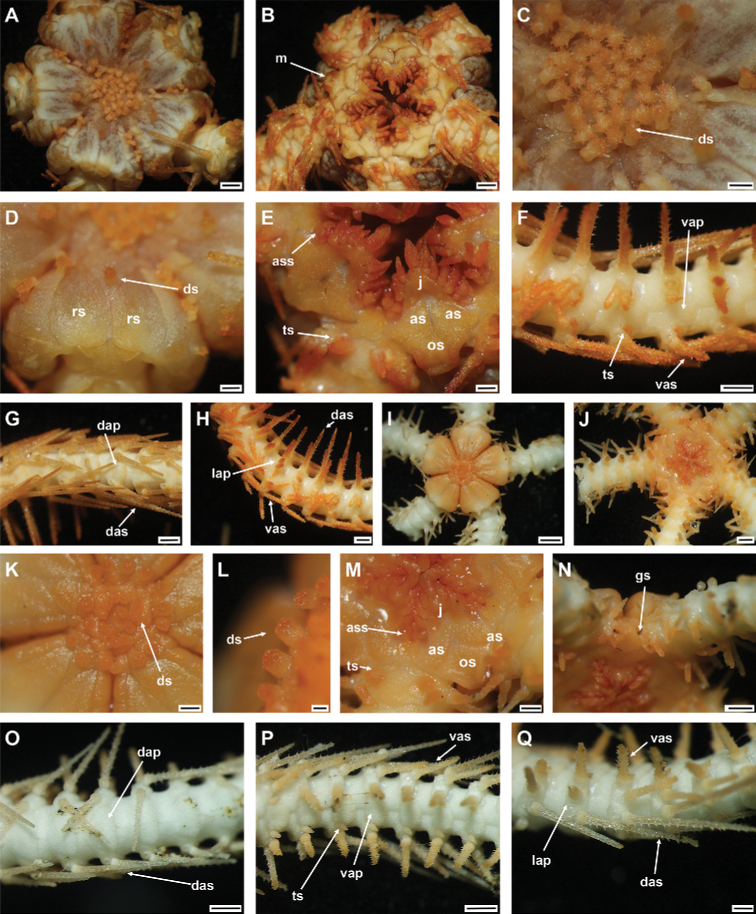
*Ophioplinthacasemele* (A. H Clark, 1949) (IDSSE-EEB-SW0113: **A–H**) **A** dorsal disc **B** ventral disc **C** center of the disc **D** radial shield **E** оral frame **F** ventral arm **G** dorsal arm **H** lateral arm; *Ophioplinthacadefensor* Koehler, 1930 (IDSSE-EEB-SW0112: **I–Q**) **I** dorsal disc **J** ventral disc **K** center of the disc **L** disc spine **M** оral frame **N** lateral disc **O** dorsal arm **P** ventral arm **Q** lateral arm. Abbreviations: **as** adoral shield, **dap** dorsal arm plate, **das** dorsal arm spine, **gs** genital slit, **j** jaw, **lap** lateral arm plate, **m** madreporite, **os** oral shield, **rs** radial shield, **ts** tentacle scale, **vap** ventral arm plate, **vas** ventral arm spine. Scale bars: 2 mm (**I**); 1 mm (**A, B, F–H, J, N–P**); 500 µm (**C–E, K, M, Q**); 200 µm (**L**).

*Ophioplinthacasemele* was first described by A. H Clark (1949) in the genus *Ophiomitra*, then redescribed by Chen et al. (2021a), and that recent study included rich morphological information. *Ophioplinthacasemele* from the present study concurs largely with the holotype description and Chen et al. (2021a), but it differs slightly in the disc stumps at the periphery of the disc. According to the holotype description, the disc stumps had a thorny tip or three thorns in the disc center, but at the periphery these stumps were smooth. Our specimen has a crown of somewhat longer thorns, both in the center and periphery of the disc (Fig. 14A–H). *Ophioplinthacadipsacos* (Lyman, 1878) is one of the species that most closely resemble *Ophioplinthacasemele* by having a similar number and shape of arm spines, radial shield characters, number of lateral oral papillae, similar disc spines, and separated dorsal and ventral arm plates, but it differs in the number of tentacle scales at the first to third tentacle pore, and in the shape of the tentacle scale (Table 3). Moreover, *O.dipsacos* was recorded from the Gulf of Mexico, far from the known distribution of *O.semele* (Lyman, 1878). *Ophioplinthacaglobata*, *O.lithosora*, *O.citata*, and *O.clothilde* show a similar shape of the disc spines, but differ in size and other morphological characters (Table 3). Therefore, variations within species from our collection can be considered as intraspecific variation, rather than species delimiting characters.

##### ﻿ Ophioplinthaca defensor

Taxon classificationAnimaliaAmphilepididaOphiacanthidae

Koehler, 1930

0AB0F3C8-019A-5AC4-8594-7264149A1E52

Fig. 14

Ophioplinthaca defensor Koehler, 1930: 84–86, pl. 9, figs1, 2; Na et al. 2021: 3–6, figs 2, 4.

###### Material examined.

Northwest Pacific • 1 specimen; near Mariana Trench, Southwest of Guam Island, seamount; 12°8.83'N, 139°0.37'E; depth 1987 m; 27 November 2020; Collecting event: stn. SC041; Shenhaiyongshi msv leg; preserved in -80°C; GenBank: OK043836; IDSSE-EEB-SW0112.

###### Distribution.

385–2000 m depth, Southwest of Guam Island, Caiwei Guyot, Weijia Guyot, Batiza Guyot, Northwest Pacific, New Caledonia, New Zealand, Tasman Sea.

###### Remarks.

*Ophioplinthacadefensor* was first described by Koehler (1930) based on a single specimen. However, Na et al. (2021) provided rich details of morphological variation from juvenile to adult *O.defensor*, and the specimen from our collection concurs with their intraspecific morphological variations (Fig. 14I–Q).

##### ﻿Genus Ophiophthalmus

Taxon classificationAnimaliaAmphilepididaOphiacanthidae

Matsumoto, 1917

EF1D8254-985B-5408-86E5-F621FA0DB5F2

###### Type species.

*Ophiacanthacataleimmoida* H. L. Clark, 1911

Included species:

*Ophiophthalmusnormani* (Lyman, 1879)

*Ophiophthalmusrelictus* (Koehler, 1904)

*Ophiophthalmushylacanthus* (H. L. Clark, 1911)

###### Diagnosis.

Adapted from Matsumoto (1917), H. L. Clark (1911), Lyman (1879), Paterson (1985), and Koehler (1904, 1922). Disc rounded to sub-pentagonal, and covered by irregular overlapping disc scales with sparse to coarse minute granules. Radial shields ovoid, naked, and widely separated by disc scales with granules. Three or four spiniform lateral oral papillae, with one ventralmost tooth at jaw apex. Dorsal arm plates contiguous at arm base then separated. Ventral arm plates pentagonal to tetragonal, and separated. Four to seven arm spines at each lateral arm plate. Arm spines smooth to rugose, one to three arm segments in length, thick, with blunt tip. Mostly single, large, flat, oval tentacle scale.

###### Distribution and habitat.

100–2194 m depth, North Pacific, Australia, New Zealand, Papua New Guinea, South Africa. Substrate of mud, fine grey sand, Foraminifera, and small stones (Olbers et al. 2019).

###### Remarks.

*Ophiophthalmus* was created by Matsumoto (1917) for species, which at the time were included in the genera *Ophiomitra*, *Ophiomitrella*, and *Ophiacantha*. However, Paterson (1985) noted that the ophiuroid genus *Ophiophthalmus* is a junior homonym of a reptilian genus described by Fitzinger (1843). Some later works (Olbers et al. 2019; Okanishi et al. 2021) used the name *Ophiophthalmus* in quotation marks, indicating its invalid status, while other works (Suppl. material 1) seem to have been oblivious to the issue, causing confusion and taxonomic instability. Article 23.9.1 of the International Code of Zoological Nomenclature (International Commission of Nomenclature 2000), states that “prevailing usage of a name must be maintained when the senior homonym has been used as a taxon’s presumed valid name, in at least 25 works, published by at least ten authors in the immediately preceding 50 years and encompassing a span of not less than ten years”. In the present case, the 50-year period extends from 1971 to 2021 and 25 publications by more than ten authors have been found in this period (Suppl. material 1).

Both names are available, because they have been published with either a description or mention of a type species, and they satisfy articles 10, 11, and 12 of the Code. Fitzinger (1843) proposed the reptile’s name *Ophiophthalmus*as a replacement name for *Lialis* Gray, 1834 with the same type species *L.burtonis* Gray, 1835, immediately making *Ophiophthalmus* Fitzinger, 1843 a junior synonym of *Lialis* (Shea 2021). Fitzinger’s contemporary colleagues and later researchers rejected his proposed name change, and *Ophiophthalmus* was thus never used for a reptile and cannot be used in the future, because it lacks a type species separate from *Lialis*. Instead, prevailing usage of the name *Ophiophthalmus* Matsumoto, 1917 has been shown here and it must be maintained.

*Ophiophthalmus* belongs to one of the largest and diverse ophiuroid families, Ophiacanthidae in the order Ophiacanthida, and is delineated from most other genera by having minute granular coverage of the disc, smooth and somewhat finely serrated arm spines, ovoid radial shields, and by characters of the arm plates (Koehler 1904, 1922; H. L. Clark 1911; Matsumoto 1917; Paterson 1985; Liao 2004; Martynov et al. 2015; Olbers et al. 2019). Currently, *Ophiophthalmus* includes four species: *O.cataleimmoidus*, *O.hylacanthus*, *O.normani*, and *O.relictus*. The genus *Ophiomitra* is closely resembles *Ophiophthalmus* by having ovoid, separated radial shields, and smooth, long arm spines, but differs in a thorny tip on granules or stumps, 10–16 oral papillae at the jaw, up to nine arm spines (Lyman 1869; Lütken and Mortensen 1899; H. L. Clark 1911; Matsumoto 1917; Koehler 1922; Olbers et al. 2019). Matsumoto (1917b) suggested that contiguous dorsal arm plates on the arm base, and the proximal arm spines not arranged in a fan shape can be used to distinguish *Ophiophthalmus* from *Ophiomitrella*, whereas Koehler (1922) distinguished these from each other by naked radial shields and overlapping disc scales, but Paterson (1985) observed that these characters are not consistent among all species within these genera. However, H. L. Clark (1911) mentioned that *Ophiophthalmus* species were remarkably consistent in some specific characters (he examined more than 4,000 specimens). Recent molecular studies suggested that *Ophiomitrella* may be polyphyletic in the family Ophiacanthidae, and species from this genus need to be revised (Christodoulou et al. 2019).

##### ﻿ Ophiophthalmus serratus sp. nov.

Taxon classificationAnimaliaAmphilepididaOphiacanthidae

DAA788C8-15CD-5F71-BDF0-38DAD310C760

http://zoobank.org/D2B3B231-FCA7-49F9-9696-328B7DD742D5

Figs 15 , 16

###### Material examined.

***Holotype*.** China • 1 specimen; South China Sea, Haima cold seep; 16°42.45'N, 110°25.68'E; depth 1378 m; 05 February 2021; Collecting event: stn. SC036; Shenhaiyongshi msv leg; preserved in 95% ethanol; GenBank: OK043837; IDSSE-EEB-SW0136. ***Paratypes*.** China • 5 specimens; South China Sea, Haima cold seep; 16°42.45'N, 110°25.68'E; depth 1378 m; 05 February 2021; Collecting event: stn. SC036; Shenhaiyongshi msv leg; preserved in 95% ethanol; GenBank: OK043838; IDSSE-EEB-SW0137 to IDSSE-EEB-SW0141. • 9 specimens; South China Sea, Haima cold seep; 16°44.02'N, 110°27.61'E; depth 1388 m; 01 May 2018; Collecting event: stn. SC036; Shenhaiyongshi msv leg; preserved in 95% ethanol; IDSSE-EEB-SW0114 to IDSSE-EEB-SW0122. • 13 specimens; South China Sea, Haima cold seep; 16°43.75'N, 110°28.34'E; depth 1378 m; 05 February 2021; Collecting event: stn. SC037; Shenhaiyongshi msv leg; preserved in 95% ethanol; IDSSE-EEB-SW0123 to IDSSE-EEB-SW0135. • 2 specimens; South China Sea, Haima cold seep; 16°34.13'N, 110°42.55'E; depth 1408 m; 07 February 2021; Collecting event: stn. SC042; Shenhaiyongshi msv leg; preserved in 95% ethanol; IDSSE-EEB-SW0142, IDSSE-EEB-SW0143.

###### Diagnosis.

**Figure 15.**
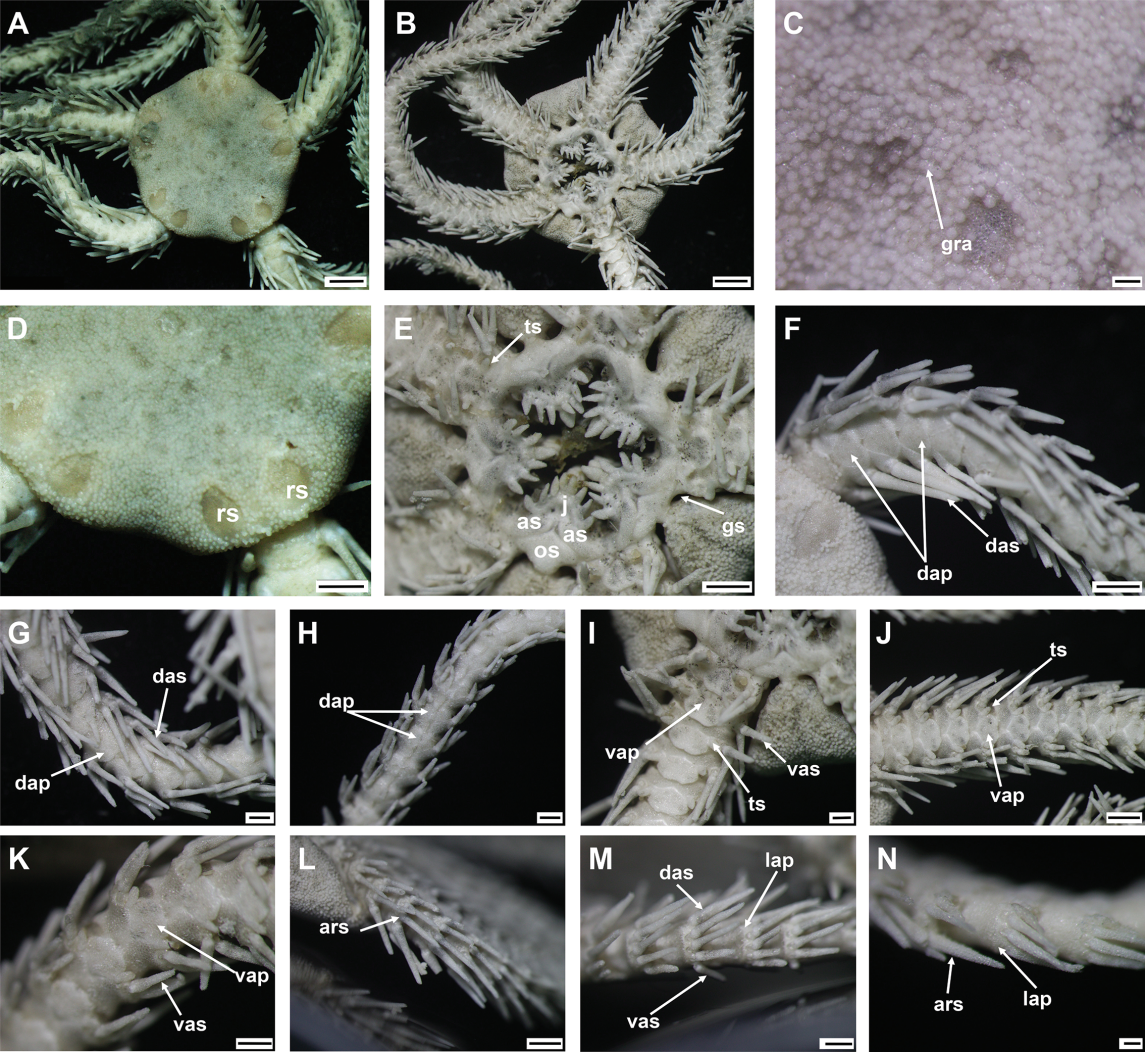
*Ophiophthalmusserratus* sp. nov., holotype (IDSSE-EEB-SW0136) **A** dorsal disc **B** ventral disc **C** center of the disc **D** radial shield **E** оral frame dorsal side of the arm base **G** dorsal arm (proximal half) **H** dorsal arm (distal half) **I** ventral side of the arm base **J** ventral arm (proximal half) **K** ventral arm (distal half) **L** lateral arm base **M** lateral arm (proximal half) **N** lateral arm (distal half). Abbreviations: **ars** arm spine, **as** adoral shield, **dap** dorsal arm plate, **das** dorsal arm spine, **gra** granules, **gs** genital slit, **j** jaw, **lap** lateral arm plate, **os** oral shield, **rs** radial shield, **ts** tentacle scale, **vap** ventral arm plate, **vas** ventral arm spine. Scale bars: 2 mm (**A, B**); 1 mm (**D–F, J, L**); 500 µm (**C, G–I, K, M**); 200 µm (**N**).

Disc circular to sub-pentagonal, covered by dense smooth granules. Radial shields ovoid, naked, and widely separated (Fig. 15A). One pointed ventralmost tooth and three slightly smaller, spiniform, finely rugose, pointed lateral oral papillae (Fig. 15E). Dorsal arm plates triangular to fan-shaped, contiguous on proximal part of arm, then separated. Five finely serrated, arm spines with blunt tip, and one slightly elongated, blunt tipped tentacle scale (Fig. 15F–I).

###### Holotype description.

Disc diameter 9.5 mm, arm base width 1.65 mm, and arm length 45–50 mm (Fig. 15).

***Disc*.** Disc circular to sub-pentagonal, raised above arm base, and covered by overlapping irregular scales, bearing rounded to cylindrical stumps with blunt tip, and smooth granules (Fig. 15A–C). Granules densely covering the surface, except radial shields, and small area in the center of the disc (Fig. 15C, D). Radial shields, ovoid, small, slightly longer than wide, naked, and widely separated (Fig. 15D). Distal edge of dorsal arm plate on arm base covered by row of few small granules, but only on two arms (Fig. 15F). Ventral disc also covered by overlapping scales with granules, but fewer granules near oral shields (Fig. 15E). Genital slits large, conspicuous, and extending from oral shield to periphery of disc (Fig. 15E). Oral shield triangular, twice as wide as long (madreporite almost as long as wide), distal end with median lobe, proximal edges straight to slightly concave, and lateral angle connected to first lateral arm plate (Fig. 15E). Adoral shields 3 × as long as wide, with straight lateral margins, and pair of shields barely connected proximally (Fig. 15E). Adoral shields connected to first lateral and ventral arm plates (Fig. 15E). Jaw large, as wide as long, with one pointed ventralmost tooth and three elongated, separated, pointed, finely rugose lateral oral papillae, slightly smaller than ventralmost tooth (Fig. 15E).

***Arms*.** Dorsal arm plates triangular to fan-shaped, twice as wide as long, distal edge slightly convex, contiguous at proximal end of arm, then separated (Fig. 15F–H). Ventral arm plate on first arm segment small, triangular, pointed distally, and slightly curved inwards proximally (Fig. 15I). Second to third ventral arm plates slightly pentagonal, wider than long, straight proximal margins, and obtuse or wavy distal edge (Fig. 15I). Following plates, as wide as long, straight lateral and proximal margins, and straight to wavy distal edge (Fig. 15J). Ventral arm plates separated along arm, including first plate (Fig. 15I–K). Lateral arm plates meeting below and above, except on dorsal arm base (Fig. 15G–N). Five finely serrated arm spines, with blunt tip in proximal to middle regions of arm, then reduced to four at distal half of arm (Fig. 15H, K, N). Dorsal arm spines one and a half to two arm segments in length (Fig. 15F, L, M). Ventral arm spines shorter, one or one and a half arm segments in length (Fig. 15J, L, M). Dorsalmost arm spine longest, next two arm spines slightly shorter, but both similar in length, and last two ventral arm spines shortest, also equal in length (Fig. 15L, M). Arm spines increasingly finely serrated to thorny, and shorter at distal end of arm (Fig. 15K, N). One slightly elongated, blunt tipped tentacle scale, nearly as long as ventral arm plate (Fig. 15I, J).

***Color*.** In ethanol, whole specimen pale brown-white. (Fig. 15).

###### Ossicle morphology of paratype.

**Figure 16.**
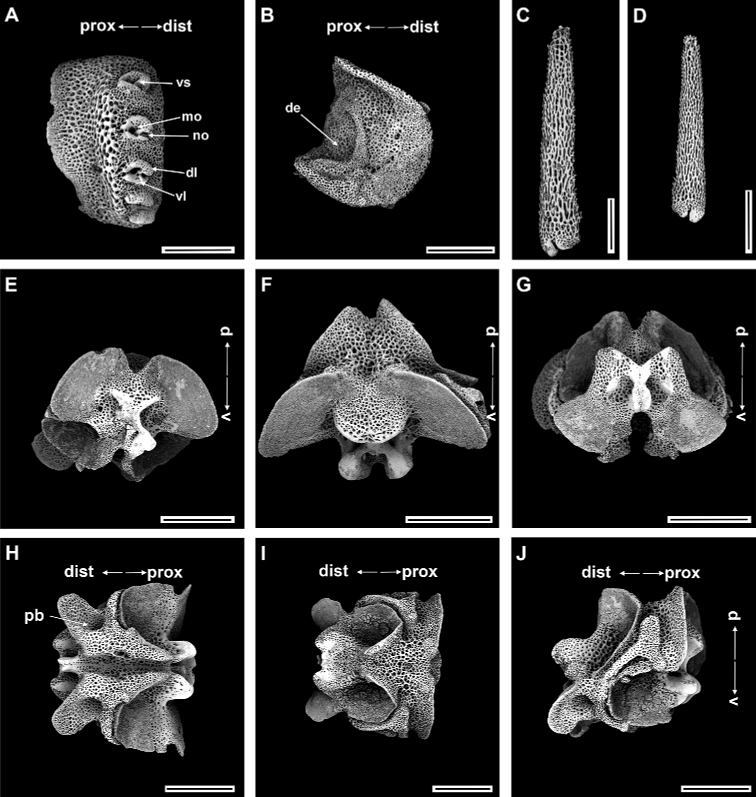
*Ophiophthalmusserratus* sp. nov., paratype (IDSSE-EEB-SW0137) **A, B** lateral arm plate **C** dorsal arm spine **D** ventral arm spine **E–J** vertebrae **E–F** proximal view **G** distal view **H** ventral view **I** dorsal view **J** lateral view. Abbreviations: **d** dorsal, **de** depression, **dist** distal, **dl** dorsal lobe, **lac** lateral ambulacral canals, **mo** muscle opening, **no** nerve opening, **pb** podial basin, **prox** proximal, **v** ventral, **vl** ventral lobe. Scale bars: 500 µm (**A–C, E–J**); 300 µm (**D**).

IDSSE-EEB-SW0137: Arm spine articulations well developed, five in number, and placed at slight angle to distal edge of lateral arm plate. Volute-shaped perforated lobe forms dorsal and distal part of articulation, but turns into two unequal subparallel curved lobes ventralwards; large muscle opening and small nerve opening (Fig. 16A). Proximal half of inner side of lateral arm plate with depression (Fig. 16B). Arm spines thorny, finely serrated with blunt apex (Fig. 16C, D). Vertebrae with streptospondylous articulation, short, broad podial basin at proximal end (Fig. 16E–J). Dorsal side of vertebrae distally triangular and proximally flattened with shallow longitudinal groove along midline (Fig. 16E–G). Ventral end of vertebrae with broad ambulacral groove with pair of lateral ambulacral canals, oral bridge absent (Fig. 16H–J).

###### Paratype variations.

Here, we examined 29 paratypes, ranging in disc diameter from 4 mm to 17 mm, and found only few notable variations among them. Large specimens had five arm spines at proximal to middle regions of the arm, then reduced to four arm spines at distal end, but small specimens showed five arm spines only at arm base, then reduced to four along the distal half of the arm. However, the finely serrated surface of the arm spine was similar in both small and large specimens. The number of lateral oral papillae differed from three to four, but most specimens had three papillae. Most specimens had dense granular coverage of the disc except larger specimens (16–17 mm). Color ranges from creamy white to dark among specimens from our collection. The above mentioned variations depend mainly on the size of the disc, and specimens with similar disc diameter showed similar morphological characters.

###### Distribution.

1378–1408 m in depth, Haima cold seep, South China Sea.

###### Etymology.

The species name was derived from the Latin word *serratus* (saw like, serrate), alluding to the surface of the arm spine.

###### Remarks.

All specimens of *Ophiophthalmusserratus* sp. nov. were collected from a methane cold seep in the South China Sea. *Ophiophthalmusserratus* sp. nov. showed similar morphological characters to three congeners, except *O.hylacanthus*. *Ophiophthalmusnormani* resembles *O.serratus* sp. nov. in having similar radial shield and arm plate characters, and granule coverage on the disc, but differs in number of arm spines (up to four), peg-like lateral oral papillae, smooth and slender arm spines, spaced granular coverage, arrangement of arm spines at lateral arm plate, and large oval tentacle scales (Lyman 1879; H. L. Clark 1911; Koehler 1922; Liao 2004). *Ophiophthalmuscataleimmoidus* is similar to *O.serratus* sp. nov. by having similar radial shield and arm plate characters, and granular coverage on the disc, but differs in number of arm spines (up to six or seven), smooth arm spines, and shape of the tentacle scales (H. L. Clark 1911; Liao 2004). *Ophiophthalmusrelictus* is similar to *O.serratus* sp. nov. by having similar radial shield and arm plate characters, and granular coverage on the disc, but differs in pointed to conical granules, six rough, short, and stout arm spines, and pointed tentacle scales (Koehler 1904; H. L. Clark 1911). *Ophiophthalmushylacanthus* is similar to *O.serratus* sp. nov. by having similar radial shield and oral frame characters, but differs significantly by rough spines on the disc, up to eight arm spines, short genital slits, and narrow, pointed tentacle scales (H. L. Clark, 1911).

## ﻿Discussion

The molecular phylogenetic analysis of the family Ophiacanthidae concurs with previous molecular studies (Christodoulou et al. 2019; O’Hara et al. 2019). In this study, we prepared a molecular phylogenetic tree of two clades that belong to the genera *Ophioplinthaca* and *Ophiophthalmus* in the family Ophiacanthidae (Fig. 2). Previous molecular studies suggested an intraspecific genetic distance of nearly 2.2% among ophiuroids, and the family Ophiacanthidae had slightly higher intraspecific and interspecific genetic distance values (Boissin et al. 2017; Christodoulou et al. 2019; O’Hara et al. 2019). However, our study showed somewhat lower genetic distance values among *Ophioplinthaca* species, probably because most of the species analyzed here live in the same biogeographic region (Northwest Pacific: *Ophioplinthacadefensor*: 0.26%; *Ophioplinthacasemele*: 0. 76%; Table 2). The phylogenetic reconstruction showed that Ophioplinthacacf.lithosora clustered with *O.globata*, whereas *O.semele* clustered with *O.plicata*, together forming sister clades among *Ophioplinthaca* species. Other sister clades were formed by *O.brachispina* sp. nov with *O.amezianeae*, and *Ophioplinthaca* sp. with *O.defensor* and *O.athena* (Fig. 2). *Ophioplinthaca* species can easily be delimited from other genera within Ophiacanthidae due to unique morphological characters, but showed highly variable, complex, and mixed morphological differences among them. Therefore, size and shape of the radial shields, and the form of the disc stumps/spines have been suggested as primary characters to delimit species of *Ophioplinthaca* (O’Hara and Stöhr 2006). *Ophioplinthaca* species from the present study were collected from the Northwest Pacific region near the Marina Trench, southwest of Guam Island, except Ophioplinthacacf.lithosora, which was collected from a South China Sea seamount. The present study and recent studies done in the Northwest Pacific region suggest higher *Ophioplinthaca* species diversity from deep seamounts than previously known, and it may increase with future expeditions to this area (Chen et al. 2021a; Na et al. 2021).

**Table 4.** Tabular key to all species of the genus *Ophiophthalmus*. Abbreviations: **ASE** arm segment, **DAP** dorsal arm plate, **DAS** dorsal arm spines, **LOP** lateral oral papillae, **RS** radial shield, **TS** tentacle scale, **VAP** ventral arm plate, **VAS** ventral arm spines, **VMT** ventralmost papillae.

Species	No. of arm spines	Radial shield	Oral frame	Tentacle scale	Dorsal arm plate (DAP) and Ventral arm plate (VAP)	Arm spine shape and length	Disc spine or granular	References
*Ophiophthalmuscataleimmoidus* (H. L. Clark, 1911)	up to 7	small, ovoid, naked, as long as wide, widely separated	3–4 LOP; 1 VMT equal of size	one, large, flat, rounded, and distinctly curved outward	1^st^VAP rounded triangular shape, then wider than long, hexagonal or pentagonal, separated DAP wider than long, triangular shape with distal curve, first few DAP with single raw of rounded grain in distal margin, contiguous only in proximal half	smooth, slender, tapering spine, next to uppermost DAS longest, 3 × ASE length	more or less sparsely with coarse, rounded granules	H. L. Clark (1911), Liao (2004)
*Ophiophthalmushylacanthus* (H. L. Clark, 1911)	up to 8	small, ovoid, naked, widely separated	3 LOP; 1 VMT, LOP smaller than VMT	one, large, flat, rounded, but become narrow and pointed along the arm	VAP wider than long, hexagonal or pentagonal, separated DAP wider than long, triangular shape with distal curve, first few DAP with rounded grain in distal margin, contiguous only at arm base	second or third form upper DAS longest more than 2 × ASE length, uppermost DAS and lowermost VAS smooth, intermediate ones with slightly rough tip	stout, pointed, rough spine, scattered coarse granules among spine near RS	H. L. Clark (1911)
*Ophiophthalmusnormani* (Lyman, 1879)	up to 4	small, ovoid, naked, as long as wide, widely separated	3 LOP, widely spaced, cylindrical, tapering, peg-like, 1 VMT	one, large and oval	1^st^VAP rounded triangular shape, then wider than long, separated DAPas wide as long, distal curve, 1–4 DAP with single raw of rounded grain in distal margin, contiguous only in proximal half	smooth, slender, blunt, and tapering spine, lowest VAS ≈ 1 × ASE, upper DAS 1–1½ × ASE length	rounded granules or short stump, sparsely spread on the disc	Lyman (1879), H. L. Clark (1911), Koehler (1922), Liao (2004)
*Ophiophthalmusrelictus* (Koehler, 1904)	up to 6	ovoid, naked, distal end well rounded, widely separated	3–4 LOP, Conical to pointed tip, 1 VMT	one, pointed	1^st^VAP rounded triangular shape, then wider than long, hexagonal or pentagonal, separated DAP wider than long, triangular shape with distal curve, DAP with rounded grain in distal margin and surface, contiguous only in proximal half	short, stout, longest one nearly 1 × ASE length, VAS quite rough near tip	dense, smooth or sometime rough minute granules	Koehler (1904), H. L. Clark (1911)
*Ophiophthalmusserratus* sp. nov.	up to 5	ovoid, naked, widely separated	3–4 LOP, rugose, pointed tip, 1 VMT	one, slightly elongated blunt tipped, as long asVAP	1^st^VAP rounded triangular shape, then slightly pentagonal, separated DAP twice as wide as long, triangular shape with distal curve, first DAP has few rounded grains in distal margin (only 2 or 3 arms), contiguous only in arm base then separated	finely serratus, blunt tip; DAS ≈ 1½–2 × ASE length, dorsalmost longest, next two similar in length, VAS shorter, 1–1½ × ASE length, rough and shorter at distal end of the arm	dense, rounded to cylindrical stumps-like smooth granules, except radial shield and small area at center of disc	this study

The species in the genus *Ophiophthalmus* share many morphological features, and the main distinguishing characters were number and shape of arm spines and maximum size. However, they have high genetic distance variations between them. As an example, the main morphological difference between *Ophiophthalmuscataleimmoidus* and *O.normani* were number of arm spines, and lateral oral papillae (Koehler 1904, 1922; H. L. Clark 1911; Matsumoto 1917; Paterson 1985; Liao 2004; Olbers et al. 2019), but they had a 21.20% high genetic distance between them. *Ophiophthalmus* species were previously recorded in the North to South Pacific Ocean, Australia, and Indonesian waters, but *Ophiophthalmusserratus* sp. nov. was the first record from the South China Sea.

## Supplementary Material

XML Treatment for Ophioplinthaca brachispina

XML Treatment for Ophioplinthaca

XML Treatment for Ophioplinthaca amezianeae

XML Treatment for Ophioplinthaca athena

XML Treatment for Ophioplinthaca cf. lithosora

XML Treatment for Ophioplinthaca semele

XML Treatment for Ophioplinthaca defensor

XML Treatment for Ophiophthalmus

XML Treatment for Ophiophthalmus serratus

## Supplementary materials

Supplementary material 1 List of 36 references that use the name *Ophiophthalmus*as a valid ophiuroid genus name between 1971 and 2021 Data type Docx file Explanation note List of 36 references that use the name *Ophiophthalmus*as a valid ophiuroid genus name between 1971 and 2021. We included the reference as evidence of prevailing usage of the name (*Ophiophthalmus*). https://binary.pensoft.net/file/682278This dataset is made available under the Open Database License (http://opendatacommons.org/licenses/odbl/1.0/). The Open Database License (ODbL) is a license agreement intended to allow users to freely share, modify, and use this Dataset while maintaining this same freedom for others, provided that the original source and author(s) are credited.Hasitha Nethupul, Sabine Stöhr, Haibin Zhang
