# Supplementary material for: ﻿Review of Ophioplinthaca Verrill, 1899 (Echinodermata, Ophiuroidea, Ophiacanthidae), description of new species in Ophioplinthaca and Ophiophthalmus, and new records from the Northwest Pacific and the South China Sea
Source: Zookeys. 2022 May 11;1099:155–202. doi: 10.3897/zookeys.1099.76479 (PMC9848918; doi:10.3897/zookeys.1099.76479)
Supplement: Supplementary material 1 — List of 36 references that use the name Ophiophthalmusas a valid ophiuroid genus name between 1971 and 2021 [file zookeys-1099-155_article-76479__-s001.docx]

**Supplement file 1.** List of 36 reference that use the name *Ophiophthalmus* as a valid ophiuroid genus name between 1971 and 2021, as evidence of prevailing usage of that name.

**References**

Allison PA, Smith CR, Kukert H, Deming JW, Bennett BA (1991) Deep-water taphonomy of vertebrate carcasses: A whale skeleton in the bathyal Santa Catalina Basin. Paleobiology 17: 78–89. [https://doi.org/10.1017/S0094837300010368](https://doi.org/10.1017/S0094837300010368%20)

Alvarado JJ, Chacón-Monge JL, Solís-Marín FA, Pineda-Enríquez T, Caballero-Ochoa AA, Rivera SS, Chaves RR (2017) Equinodermos del museo de zoología de la universidad de Costa Rica. Revista de Biologia Tropical 65: S272–S287. [https://doi.org/10.15517/rbt.v65i1-1.31695](https://doi.org/10.15517/rbt.v65i1-1.31695%20)

Baker AN (1979) Some Ophiuroidea from the Tasman Sea and adjacent waters. New Zealand Journal of Zoology 6: 21–51.

Bennett BA, Smith CR, Glaser B, Maybaum HL (1994) Faunal community structure of a chemoautotrophic assemblage on whale bones in the deep northeast Pacific Ocean. Marine Ecology Progress Series 108: 205–224. [https://doi.org/10.3354/meps108205](https://doi.org/10.3354/meps108205%20)

Bozzano A, Sardaà F (2002) Fishery discard consumption rate and scavenging activity in the northwestern Mediterranean Sea. ICES Journal of Marine Science 59: 15–28. [https://doi.org/10.1006/jmsc.2001.1142](https://doi.org/10.1006/jmsc.2001.1142%20)

Carey AG (1990) Distributional Ecology of Selected Megaepifauna on Abyssal Plains Adjacent to Gorda Ridge, Northeast Pacific Ocean BT - Gorda Ridge. In: McMurray GR (Ed.), Springer New York, New York, NY, 241–251.

Carey AG, David Jr. LS, Taghon GL, Anne ED (1988) 88 National undersea research program Biology and ecology of the Oregon continental shelf edge associated with the accretionary prism.

Clark AM (1977) The South African Museum’s Meiring Naude Cruises, Part 4. Echinoderms. Annals of the South African Museum 73: 133–147.

Hobson KA, Fisk A, Karnovsky N, Holst M, Gagnon JM, Fortier M (2002) A stable isotope (δ13C, δ15N) model for the North Water food web: Implications for evaluating trophodynamics and the flow of energy and contaminants. Deep-Sea Research Part II: Topical Studies in Oceanography 49: 5131–5150. [https://doi.org/10.1016/S0967-0645(02)00182-0](https://doi.org/10.1016/S0967-0645(02)00182-0%20)

Kemp KM, Jamieson AJ, Bagley PM, McGrath H, Bailey DM, Collins MA, Priede IG (2006) Consumption of large bathyal food fall, a six month study in the NE Atlantic. Marine Ecology Progress Series 310: 64–76. [https://doi.org/10.3354/meps310065](https://doi.org/10.3354/meps310065%20)

Lampitt RS, Billett DSM, Rice AL (1986) Biomass of the invertebrate megabenthos from 500 to 4100 m in the northeast Atlantic Ocean. Marine Biology 93: 69–81. [https://doi.org/10.1007/BF00428656](https://doi.org/10.1007/BF00428656%20)

Grygier Mark J. (1987) Nauplii, Antennular ontogeny, and the position of the Ascothoracida within the Maxillopoda. Jounal of Crustacean Biology 1: 87–104.

Masanori Okanishi (2020) Ophiuroids from the Kumano Sea. Journal of the Japanese Zoological Society 48: 20–26. [https://doi.org/10.19004/taxa.48.0_20](https://doi.org/10.19004/taxa.48.0_20%20)

McKnight DG (1993) Records of echinoderms (Excluding holothurians) from the Norfolk Ridge and Three Kings Rise north of New Zealand. New Zealand Journal of Zoology 20: 165–190. [https://doi.org/10.1080/03014223.1993.10422858](https://doi.org/10.1080/03014223.1993.10422858%20)

McKnight DG, Probert PK (1997) Epibenthic communities on the Chatham Rise, New Zealand. New Zealand Journal of Marine and Freshwater Research 31: 505–513. [https://doi.org/10.1080/00288330.1997.9516784](https://doi.org/10.1080/00288330.1997.9516784%20)

Metaxas A, Giffin B (2004) Dense beds of the ophiuroid *Ophiacantha abyssicola* on the continental slope off Nova Scotia, Canada. Deep-Sea Research Part I: Oceanographic Research Papers 51: 1307–1317. [https://doi.org/10.1016/j.dsr.2004.06.001](https://doi.org/10.1016/j.dsr.2004.06.001%20)

Morales-Zárate MV, Zayas-Álvarez A, Salinas-Zavala CA, Mejía-Rebollo A (2016) Biocenosis de la comunidad bentónica en la Laguna Guerrero Negro, Baja California Sur, México: Caracterización espacio-temporal. Latin American Journal of Aquatic Research 44: 726–741. [https://doi.org/10.3856/vol44-issue4-fulltext-8](https://doi.org/10.3856/vol44-issue4-fulltext-8%20)

O’Hara TD (2008) 66 Journal of Vocational Behavior Bioregionalisation of Australian waters using brittle stars (Echinodermata:Ophiuroidea), a major group of marine benthic. 1–69 pp.

O’Hara TD, Rowden AA, Williams A (2008) Cold-water coral habitats on seamounts: Do they have a specialist fauna? Diversity and Distributions 14: 925–934. [https://doi.org/10.1111/j.1472-4642.2008.00495.x](https://doi.org/10.1111/j.1472-4642.2008.00495.x%20)

Ohmura T, Hamatsu T, Yamauchi M, Takahashi T (2006) Spatial distribution patterns and nutritional status of kichiji rockfish *Sebastolobus macrochir* in summer on the continental slope off the Pacific coast of Hokkaido, Japan. Nippon Suisan Gakkaishi (Japanese Edition) 72: 430–439. [https://doi.org/10.2331/suisan.72.430](https://doi.org/10.2331/suisan.72.430%20)

Piepenburg D (2000) Arctic brittle stars (Echinodermata: Ophiuroidea). Oceanography and marine biology 38: 189–256.

Piepenburg D, Blackburn TH, vonDorrien CF, Gutt J, Hall PO, Hulth S, Kendall MA, Opalinski KW, Rachor E, Schmid MK (1995) Partitioning of benthic community respiration in the Arctic (northwestern Barents Sea). Marine Ecology Progress Series 118: 199–214. [https://doi.org/10.3354/meps118199](https://doi.org/10.3354/meps118199%20)

Siebenaller JF (2010) Effects of the Deep-Sea Environment on Invertebrates. 1st ed. 59 pp.

Smith CR (1985) Food for the deep sea: utilization, dispersal, and flux of nekton falls at the Santa catalina basin floor. Deep Sea Research Part A, Oceanographic Research Papers 32: 417–442. [https://doi.org/10.1016/0198-0149(85)90089-5](https://doi.org/10.1016/0198-0149(85)90089-5%20)

Smith CR (1986) Nekton falls, low-intensity disturbance and community structure of infaunal benthos in the deep sea. Journal of Marine Research 44: 567–600. [https://doi.org/10.1357/002224086788403015](https://doi.org/10.1357/002224086788403015%20)

Smith CR, Hamilton SC (1983) Epibenthic megafauna of a bathyal basin off southern California: patterns of abundance, biomass, and dispersion. Deep Sea Research Part A, Oceanographic Research Papers 30: 907–928. [https://doi.org/10.1016/0198-0149(83)90048-1](https://doi.org/10.1016/0198-0149(83)90048-1%20)

Smith KL (1983) Metabolism of two dominant epibenthic echinoderms measured at bathyal depths in the Santa Catalina Basin. Marine Biology 72: 249–256. [https://doi.org/10.1007/BF00396830](https://doi.org/10.1007/BF00396830%20)

Smith KL, Brown NO (1983) Oxygen consumption of pelagic juveniles and demersal adults of the deep-sea fish Sebastolobus altivelis, measured at depth. Marine Biology 76: 325–332. [https://doi.org/10.1007/BF00393036](https://doi.org/10.1007/BF00393036%20)

Smith KL, Carlucci AF, Jahnke RA, Craven DB (1987) Organic carbon mineralization in the Santa Catalina Basin: benthic boundary layer metabolism. Deep Sea Research Part A, Oceanographic Research Papers 34: 185–211. [https://doi.org/10.1016/0198-0149(87)90081-1](https://doi.org/10.1016/0198-0149(87)90081-1%20)

Thompson BE (1980) New Bathyal Sipunculan From Southern California, With Ecological Notes. Deep-Sea Research, Part A: Oceanographic Research Papers 27: 951–957. [https://doi.org/10.1016/0198-0149(80)90006-0](https://doi.org/10.1016/0198-0149(80)90006-0%20)

Toshihiko Fujita (2018) Natural history science of echinoderms and its promotion in Japan. Proceeding of the Japanese Society of Systematic Zoology 45: 4–15.

Wheatcroft RA (1991) Conservative tracer study of horizontal sediment mixing rates in a bathyal basin, California borderland. Journal of Marine Research 49: 565–588. [https://doi.org/10.1357/002224091784995792](https://doi.org/10.1357/002224091784995792%20)

Wheatcroft RA (1992) Experimental tests for particle size‐dependent bioturbation in the deep ocean. Limnology and Oceanography 37: 90–104. [https://doi.org/10.4319/lo.1992.37.1.0090](https://doi.org/10.4319/lo.1992.37.1.0090%20)

Wheatcroft RA, Smith CR, Jumars PA (1989) Dynamics of surficial trace assemblages in the deep sea. Deep Sea Research Part A, Oceanographic Research Papers 36: 71–91. [https://doi.org/10.1016/0198-0149(89)90019-8](https://doi.org/10.1016/0198-0149(89)90019-8%20)

Witte U (1999) Consumption of large carcasses by scavenger assemblages in the deep Arabian Sea: Observations by baited camera. Marine Ecology Progress Series 183: 139–147. [https://doi.org/10.3354/meps183139](https://doi.org/10.3354/meps183139%20)

Witte U, Graf G (1996) Metabolism of deep-sea sponges in the Greenland-Norwegian sea. Journal of Experimental Marine Biology and Ecology 198: 223–235. [https://doi.org/10.1016/0022-0981(96)00006-8](https://doi.org/10.1016/0022-0981(96)00006-8%20)
